# Painterly depiction of material properties

**DOI:** 10.1167/jov.20.7.7

**Published:** 2020-07-07

**Authors:** Mitchell J. P. van Zuijlen, Sylvia C. Pont, Maarten W. A. Wijntjes

**Affiliations:** Perceptual Intelligence Lab, Industrial Design Department, Delft University of Technology, Delft, the Netherlands; Perceptual Intelligence Lab, Industrial Design Department, Delft University of Technology, Delft, the Netherlands; Perceptual Intelligence Lab, Industrial Design Department, Delft University of Technology, Delft, the Netherlands

**Keywords:** material perception, material attributes, art history, Amazon Mechanical Turk

## Abstract

Painters are masters of depiction and have learned to evoke a clear perception of materials and material attributes in a natural, three-dimensional setting, with complex lighting conditions. Furthermore, painters are not constrained by reality, meaning that they could paint materials without exactly following the laws of nature, while still evoking the perception of materials. Paintings have to our knowledge not been studied on a big scale from a material perception perspective. In this article, we studied the perception of painted materials and their attributes by using human annotations to find instances of 15 materials, such as wood, stone, fabric, etc. Participants made perceptual judgments about 30 unique segments of these materials for 10 material attributes, such as glossiness, roughness, hardness, etc. We found that participants were able to perform this task well while being highly consistent. Participants, however, did not consistently agree with each other, and the measure of consistency depended on the material attribute being perceived. Additionally, we found that material perception appears to function independently of the medium of depiction—the results of our principal component analysis agreed well with findings in former studies for photographs and computer renderings.

## Introduction

Materials represent the “stuff’ that things are made of (Adelson, 2001). We interact daily with these “things,” either physically (e.g., manual interaction) or visually (e.g., assessing ripeness, quality, or value). While the importance of material perception for humans seems evident, we lack a full understanding of the underlying mechanisms. In a previous study, Fleming, Wiebel, and Gegenfurtner (2013) investigated the relationships between attribute ratings and material classes (e.g., wood, glass, foliage, etc.) for photographs. In this article, we extended on this study by using a big date data approach to measure the perception of material properties in paintings. Our investigation is motivated by the assumption that to depict materials convincingly, painters presumably hold insights into visual cues that lead to the perception of various attributes.

Painters are masters of depiction and are capable of evoking a clear perception of a three-dimensional (3D) world, with complex lighting and recognizable materials. Interestingly, although the appearances of real materials are limited by the rules of physics, materials as depicted in paintings have no such constraints. Incongruencies between paintings and reality often go unnoticed by the viewer (Cavanagh, 2005). Instead of strictly following physics, painters have extracted the essential visual cues needed to trigger the perception of materials. Di Cicco, Wijntjes, and Pont (2019) studied visual cues for gloss, which were implicitly discussed in a painting manual by the seventeenth-century painter Willem Beurs (Beurs, 1692). They found that predictors that explained a large portion of the variance in gloss perception had implicitly been described within this seventeenth-century manual. This shows that painters held insights into perception and that studying art could lead to new insights for perception scientists.

While art reveals insights into perception, conversely perception can be used to understand art. For example, several important art historical publications (Arnheim, 1941; Gombrich, 1960; Baxandall, 1995) use knowledge about perception to analyze art. Anecdotally, this approach can also be seen in artistic attributions such as in the case of *Still Life with Grapes and a Bird*, which is attributed to Antonio Leonelli by the Metropolitan Museum curator. In his attribution, the curator comments on “The tendency to geometrize the forms with shading that rigorously enhances their rotundity […] the emphasis on surface effects—the grained wood […] the clearly delineated shadows.” It is interesting to see that many of the curator's terms are conceptually very similar to those used in perception science. The overlap between the perceptual sciences and art means that a fuller understanding of perceptual concepts could be beneficial for both fields. Yet, how to study and quantify the depiction of materials in paintings? There are several standard psychophysical methods that potentially apply to the study of depicted materials, such as matching tasks, similarity ratings, or attribute ratings. The first method requires a material probe, which is an interactive image that can be adjusted to match the material attributes of the target stimulus. The probe can be parameterized by an analytical physical model (e.g., Ward), weight parameters of data-driven Bidirectional Reflectance Distribution Functions (BRDFs) (Matusik, Pfister, Brand, & McMillan., 2003) or additive mixing of basis images representing canonical modes (Griffin, 1999; Zhang, de Ridder, Fleming, & Pont, 2016). As such, material matching tasks require predefined models for each material or sets of basis (BRDF) samples or (canonical mode) images to represent a wide range of materials. These methods are suitable for testing a wide range of materials, but not for all materials. For example, varying 3D textures (based on bidirectional texture functions [BTFs]) systematically and fluently is technically extremely hard. Moreover, there is no method yet to vary 3D textures in a tractable interface such that all materials are covered. Therefore material matching is not suited to study the wide variety of material attributes found in paintings as we aim to do here.

A second method, similarity ratings, relies on systematic variations of the stimulus set. Pellacini, Ferwerda, and Greenberg (2000) asked participants to rate the apparent difference in gloss between pairs of images, without defining gloss. They then used multidimensional scaling to infer the dimensionality of gloss. Often, the similarity is not specified to the observer and can comprise any combination of subjective criteria. Radonjić, Cottaris, & Brainard (2015) asked participants to judge which of two test patches rendered under varying illuminations was more similar to a third patch under a fixed illumination, to investigate the relative contribution of illumination on color-constancy. The fact that comparisons are made between pairs or triplets implies that a very large number of trials (i.e., quadratically increasing with sample size for pairs) is needed. For the large number of materials that we aim to study, this method is thus not feasible.

Last, a popular method relies on attribute scaling. In this method, a participant either rates single images explicitly (e.g., how glossy is this material?) or makes implicit forced-choice pairwise comparisons (e.g., which of these two images is glossier?). However, making comparisons inflates the trial number, so we decided to choose attribute ratings for single images to study materials depicted within paintings. This raises a straightforward question: which attribute names should be chosen that most completely covers the perception of the wide variety of materials present in paintings?

Whereas a large variety of attributes has been investigated previously in perception literature, the majority of these attributes are studied in isolation, such as glossiness (Chadwick & Kentridge, 2015; Wiebel, Toscani, & Gegenfurtner, 2015; Ferwerda, Pellacini, & Greenberg, 2001; Wijntjes & Pont, 2010; Kim, Marlow, & Anderson, 2012; Marlow & Anderson, 2013), translucency (Fleming & Bulthoff, 2005; Motoyoshi, 2010; Xiao et al., 2014) or transparency (Nakayama, Shimojo, & Ramachandran, 1990; Motoyoshi, 2010; Fleming, Jäkel, & Maloney, 2011). These studies often investigate how the perception of attributes are affected by various distal cues such as shape (Fleming, Torralba, & Adelson, 2004; Marlow & Anderson 2015) and light (Fleming, Dror & Adelson, 2003; Adams, Graf, & Ernst, 2004) or proximal (image structure) cues (Motoyoshi, Nishida, Sharan, & Adelson. 2007; Sharan, Motoyoshi, Nishida, & Adelson, 2008; Marlow & Anderson, 2013).

Perceptual attributes are also studied in computer science, albeit with different motivations. Because attributes—such as gloss, translucency, and roughness—seem to be intuitive, perceptual parameters (i.e., attributes) are often preferred over physical parameters for rendering interfaces. To develop intuitive interfaces Serrano, Gutierrez, Myszkowski, Seidel, & Masia (2018) collected attribute ratings for 14 material attributes, also including high-level class descriptors such as plastic-like, fabric-like, and metallic-like. They mapped these perceptual attributes to an underlying principal component analysis (PCA)–based representation of BRDFs (i.e., physical parameters) and showed that their functionals were good predictors of the perceived material attributes.

Aside from perception and graphics, industrial design also makes use of quantitative attribute descriptions of materials. Designers use attributes to investigate and design user experiences (Karana, Hekkert & Kandachar, 2009). Interestingly, these three disciplines use partially overlapping but also distinct vocabularies to describe attributes. In computer science (e.g., Matusik et al., 2003; Serrano et al., 2018) a large portion of attributes refer to material classes, such as metallic-like, plastic-like, ceramic-like, while perception and design studies often focus more on sensorial qualities like fragility, hardness, and elasticity. The material classes in our study were based on human annotations. The attributes we selected for our study are mostly based on the perception and design studies. We arrived at a set of 15 materials and 10 attributes, the details of which will be explained in the methods section.

In this article, we studied perceived material classes and how they vary in perceived material attributes for a large set of paintings. We first present our methods relatively extensively, as this partly consisted of collecting the painting images. Furthermore, we detail how we collected a large set of annotated segmentations of paintings via online experiments. Then we present the results, first addressing the subjects’ consistency, as validation of our method, followed by a detailed analysis of the collected material judgments.

## Methods

Our stimulus collection serves a broader goal than the study reported here. The collection and annotation of artworks is part of ongoing research that will be comprehensively published at a later stage. In the current study, we perform a perceptual experiment in which we use a subset of the artworks and annotations we collected. We nevertheless report all the details on the collection of data for sake completeness.

In the following paragraphs, we detail our artwork annotation pipeline. This is followed by the perception experiment, in which participants judged material attributes for various material classes.

### Ethics

The study conformed to the declaration of Helsinki and was approved by the ethical review committee of the Technical University of Delft. All data were collected anonymously.

### Stimulus collection

In the context of a project where we are creating a database of depicted materials and their properties, we collected material segments from paintings. Because this process was not part of the current studies’ scope, we report it in the supplementary material. Below we report a summary.

We created a list of materials, based on the previous research mentioned in the introduction, plus observations of the paintings and our desire to cover as many materials in those paintings as possible. The list contains 15 materials:

**Table:** 

• Animal	• Gem	• Paper*
• Ceramic	• Glass*	• Skin
• Fabric*	• Ground	• Sky
• Flora†	• Liquid†	• Stone*
• Food	• Metal*	• Wood

The material list has six items in common with the Flickr Material Database (FMD; Sharan, Rosenholtz and Adelson, 2009; Sharan, Liu, Rosenholtz, & Adelson, 2013). These common items have been indicated above with an asterisk (*). Additionally, two materials from the FMD, foliage and water, were incorporated within our list as part of our broader labels flora and liquids—indicated with a dagger (†). These eight materials were also used by Fleming et al. (2013) with the original names as defined in the FMD. The remaining seven materials were included for a variety of reasons. Animal and food are two instances of materials that we included as an overarching concept, encompassing many different materials such as fruits, vegetables, and bread for food, and materials such as fur, claws, feathers, and scales for animal. We included gem to contain items such as pearls and precious stones. Ground and Sky were included because they often cover large portions of the painting's surface. Note that (1) we defined ground as things such as dirt and gravel, without grasses or shrubbery, because those should be identified as flora and (2) we counted clouds as belonging to the sky. Last, we included (human) skin, instead of an overarching human concept such as is done with animal and food. We made this decision because skin is a very interesting material in its own right (sometimes even referred to as the “holy grail” of rendering) both from a classic perceptual point of view (Stephen, Coetzee, & Perrett 2011; Matts Fink, Grammer, & Burquest, 2007), as well as from a computer science perspective (Igarashi, Nishino, & Nayar, 2017; Jensen, Marschner, Levoy, & Hanrahan, 2001) and an art-historical point of view (Lehmann, 2008).

#### Annotation pipeline

In 2013, Bell, Upchurch, Snavely, & Bala published OpenSurfaces, a database with annotated and segmented materials. This database is a public resource and is available at http://opensurfaces.cs.cornell.edu/. Bell et al., (2013) created this database to fill the need within computer graphics to accurately model materials within context. Besides the database, they made their annotation pipeline, that is, their process of collecting data, open-source. We have adapted their annotation pipeline to fit our purposes for the collection of material segmentations and annotations.

#### Collecting stimuli

The collection of stimuli was executed in multiple steps. Here we provide a summary of each step. Each step is discussed in-depth within the supplementary materials. Step 1, collecting paintings: we collected digital images and the associated meta-data for paintings from seven online museum galleries. Step 2, collecting materials: we used AMT to measure inferences of what materials were depicted within the painting. Participants had to indicate which paintings contained a requested material. For each painting, we collected at least five responses for each material and required an agreement of 80% to consider a painting to contain the material. Step 3, segment collection: participants segmented materials from paintings. In each task, the participant would see a painting and be requested to segment one instance of a specific material that was indicated to be present within the painting in step 2. Step 4, quality check: the quality of the created segments was checked by a minimum of 5 participants. Step 5, material check: It is possible that a participant wrongfully segmented *wood*, when tasked with segmenting *metal*; therefore in this step we asked participants to indicate what they perceived the material of the segment to be. Step 6, manual selection: in the end, we manually selected the 90 best segments per material.

### Perceptual experiment

Using the selected segments discussed above, we had a total of 198 AMT participants rate 10 perceptual attributes for each of these segments. The perceptual attributes are listed below. All participants were located within the United States, according to AMT, and each participant had previously completed at least 1000 tasks on the AMT platform, of which at least 95% had been accepted by the creators of those tasks.

#### Attributes and image statistics

We created a list of 10 perceptual material attributes. Our attribute list has five items in common with Fleming et al. (2013), that is, those indicated with an asterisk. When applicable we have copied the original attribute definitions and we have created our attribute definitions to be similar to the other attributes used in Fleming et al. (2013). Additionally, we split colorful into multicolored and vivid. We expected colorful might be difficult for naïve participants, because it could be interpreted as “many, low-intensity colors” or “a single, very intense color” for multicolored and vividness, respectively. Additionally, we added translucent to the existing transparent attribute, because we found that some participants were aware of the optically defined difference between these two, whereas some were not. Altogether this resulted in the following list and definitions:

• Bendable: How bendable is the material? Low values indicate that the material is highly rigid and could not easily be bend; high values indicate that a small force would be required to bend the material.
• Cold*: To what extent would you expect the surface to feel cold to the touch? Low values indicate that the material would typically feel warm or body temperature; high values indicate that the material would feel cold to the touch.
• Fragile*: How fragile or easy to break is the material? Low values indicate that the material is highly resistant and could not easily be broken; high values indicate that a small amount of force would be required to break, tear, or crumble the material.
• Glossy*: How glossy or shiny does the material appear to you? Low values indicate a matte, dull appearance; high values indicate a shiny, reflective appearance.
• Hairy: If you were to reach out and touch the material, how hairy would it feel? Low values indicate that the surface would feel hairless; high values indicate that it would feel hairy.
• Hard*: If you were to reach out and touch the material, how hard or soft would it feel? How much force would be required to change the shape of the material? Low values indicate that the surface would feel soft; high values indicate that it would feel hard.
• Multicolored: How multi-colored does the material appear to you? Low values indicate a monochrome (single-colored) appearance; high values indicate many colors.
• Rough*: If you were to reach out and touch the material, how rough would it feel? Low values indicate that the surface would feel smooth; high values indicate that it would feel rough.
• Transparent/translucent: To what extent does the material appear to transmit light? Low values indicate an opaque appearance; high values indicate the material allows a lot of light to pass through it.
• Vivid: How vivid does the material appear to you? Low values indicate a dull, grayish appearance; high values indicate a strong vivid color.

Next, we also defined and calculated four simple, image histogram statistics for each of the material segments.

• The contrast: Defined as the Michelson contrast: $contrast\;=\frac{L_{max}-L_{min}}{L_{max}+L_{min}}\;,w$here *L_max_* and *L_min_* are taken as the ninety-fifth and fifth percentile of the luminance distribution of the material segment (Michelson, 1891).
• Skewness: The skewness of the luminance distribution of the material segment.
• Colorful: The colorfulness, measured as the ratio of voxels filled in 3D RGB color space to the total number of voxels, where the RGB color space was rescaled to 0 to 15, as opposed to the conventional 0 to 255, for each of the material segments.
• Mean luminance: the mean luminance of the image segment.

#### Stimuli

From the 90 segments per material—as discussed above—we randomly selected 30 segments for each of the 15 materials, making a total of 450 stimuli. We chose to include this randomization to reduce the chance of experimenter bias, considering we originally selected the 90 segments per material. We subdivided these 450 segments into five sets of 90, where each set contained six segments per material. In other words, each set had six segments of *wood,* six segments of *metal,* and more. These sets were used in experimental blocks. We chose to partition the data into these five sets, to reduce the number of trials per participant. Without partitioning the data into these 5 sets, every participant would have needed to complete (450 stimuli × 3 repetitions =) 1350 trials, which we consider too many for web-based experiments. With these five sets, participants only need to complete 270 trials. The specific choice of five sets, over, for example, nine sets, is arbitrary. Splitting the experiments into these sets implies that we calculated interrater reliability within each set.

**Figure 1.**
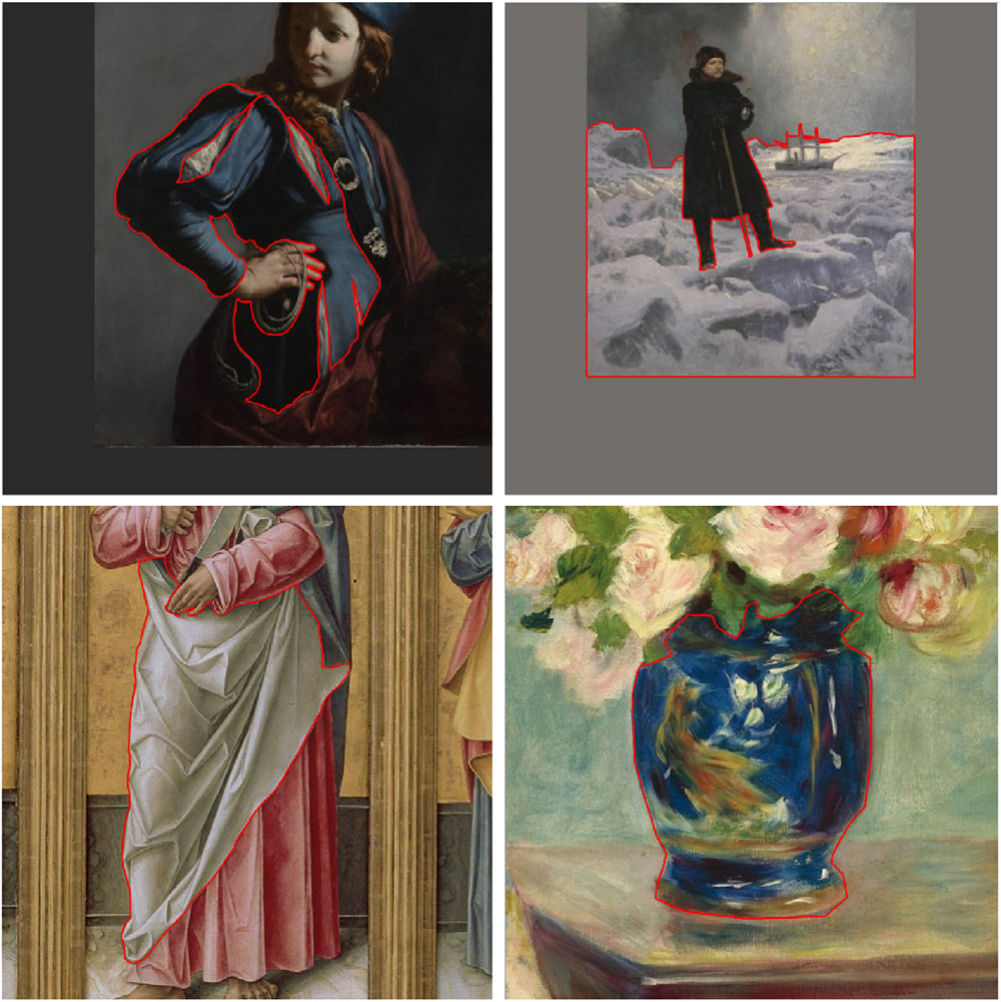
Examples of four stimuli. For the top two, the context size exceeds the dimensions of the original painting, and the overflow has been colored with the average RGB color value of the painting contained within the bounding box. For the bottom two, the context size does not exceed the original painting dimensions and is thus only a section of the painting without any overflow. The red outlines indicate the segments. From top-left to bottom-right: detail of David with the Head of Goliath (c. 1645) by Guido Cagnacci; The Explorer A.E. Nordenskiöld (1886) by Georg von Rosen; detail of Polyptych with Saint James Major, Madonna and Child, and Saints (1490) by Bartolomeo Vivarinil; and detail of Mlle Charlotte Berthier (1883) by Auguste Renoir.

We presented the segments in a section of the original painting. We created a square *context box* around the segment, which is, in essence, a bounding box around the segment with margin. The *context box* size was calculated as the maximum of the width or height of the segment, multiplied by 1.25. We took the maximum to ensure the *context box* is a perfect square. In some cases, this meant that the *context box* boundaries exceeded the dimensions of the original painting. To keep the aspect ratio consistent, we included this overflow as part of the segment and colored the overflow with the average of the color of the painting part within the bounding box. A few examples can be seen in Figure 1.

#### Procedure

**Figure 2.**
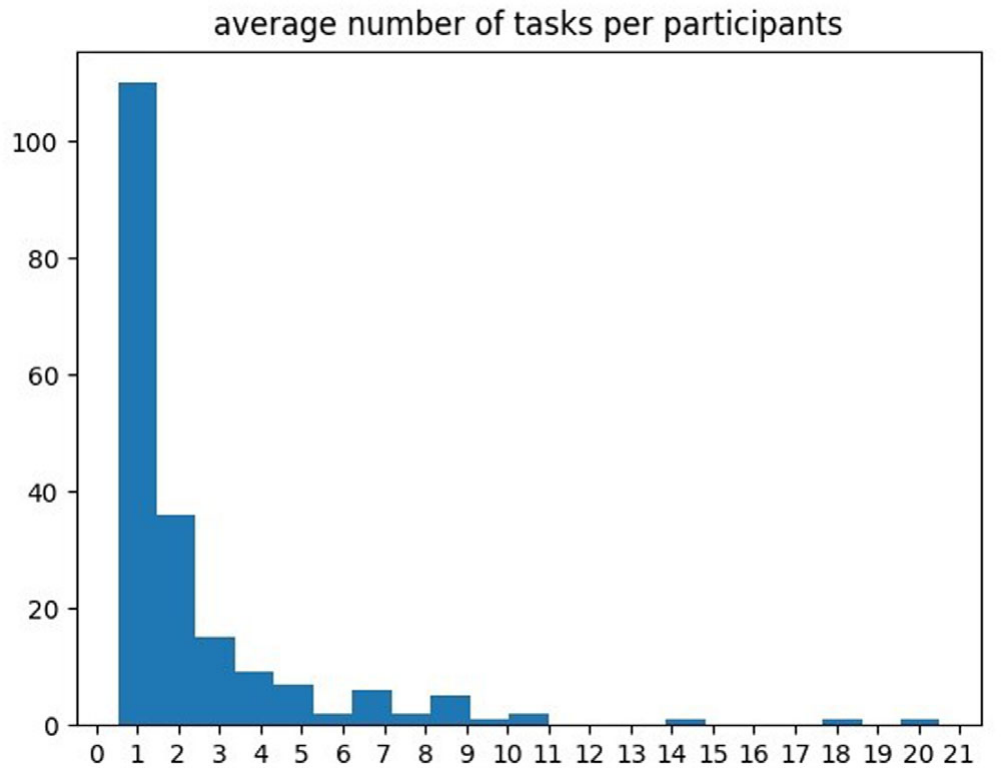
Distribution of completed rating tasks per participants.

Each of the five sets of images was rated on each of the 10 attributes, making a total of 50 set/attribute combinations. Each of 50 combinations would be rated by 10 different participants. Each participant would only see one set of images and rate this set on one attribute per task. Participants could choose how many of these combinations they would rate. This means that a single participant could, in theory, do each of the 50 experimental blocks once and that the total number of participants should be between 10 (i.e., each participant did all 50 set/attribute combinations) and 500 (i.e., each participant did only one task). In practice, 198 participants performed the task on average 2.5 times each, with 110 participants only performing one task. The full distribution is presented in Figure 2.

Each task contained three repetitions of the 90 images, making a total of 270 trials. We used a Fisher-Yates Shuffle to create three shuffled permutations of the set and concatenated these three permutations in each task. This allows us to measure the intraobserver correlation (with three repetitions) next to the interobserver correlations (with 10 repetitions)

#### Task

Participants on the AMT platform were capable of choosing and selecting what tasks they wanted to work on. Once participants had selected our tasks, they would first be shown a text-based tutorial. After a 10-second interval, participants were able to start the task. First, the tasks displayed the main question in bold: “How [attribute] is this material?,” followed by our definition of that attribute. To give participants an impression of the range of stimuli we showed them a random selection of one-third of the stimuli, on which they were told to base their ratings. They could click the *start* button to start the first trial.

**Figure 3.**
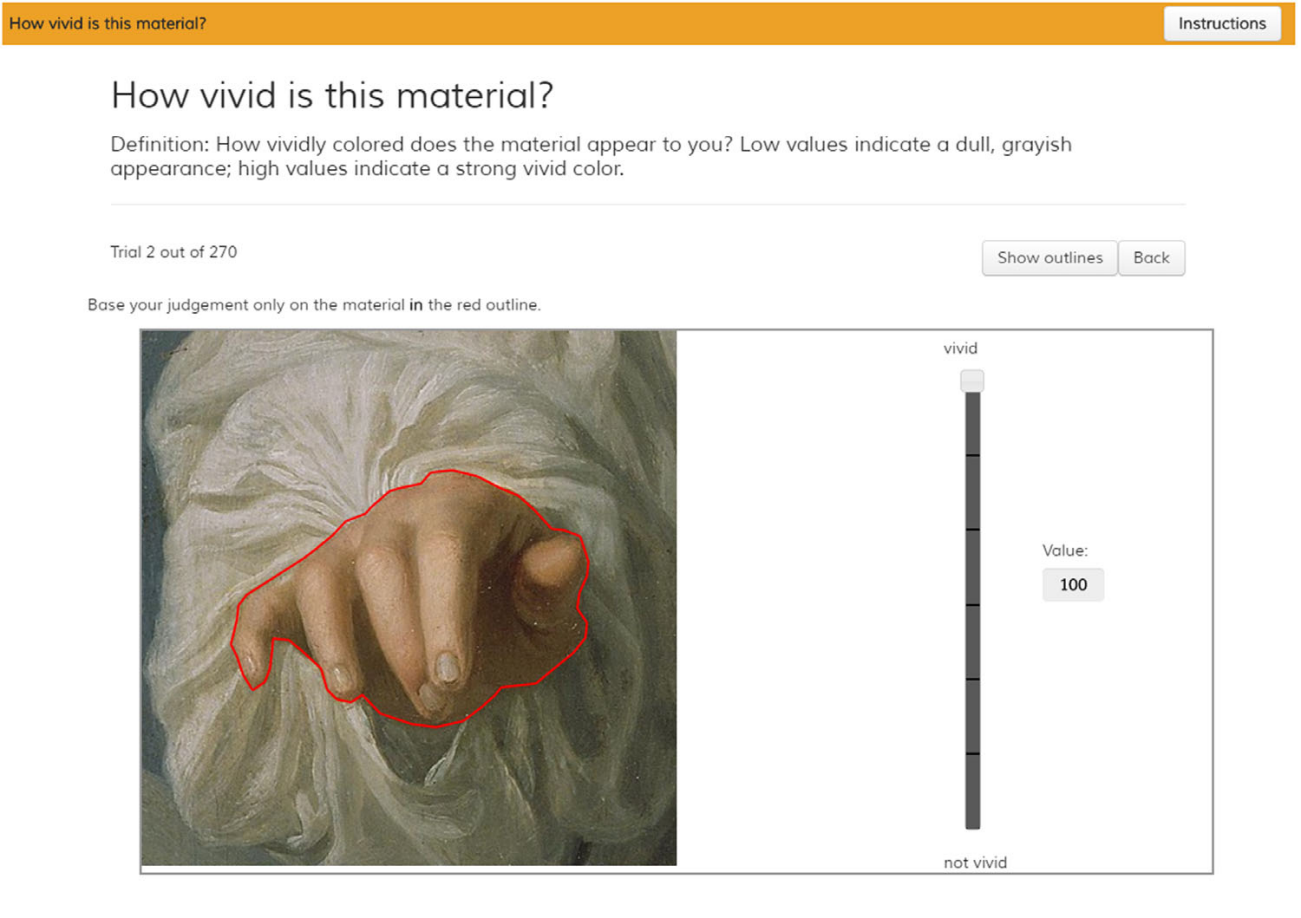
Example of the perceptual judgment task. At the top, the question and definition are repeated, which participants would have seen in the instructions. The task shows one segment at a time, as part of the original painting. In the live version, the red outline appears flashing at around 10 hz at the onset of each trial (or when the participant pressed the corresponding button) to indicate the segment boundaries and disappears after a second. The slider can be moved by moving the mouse up and down, whereas a left mouse-click progresses the experiment to the next trial. The painting is a section of The Annunciation (c.1660) by Godfried Schalcken.

In each trial, the participants were shown the same question and definition as mentioned above, as well as one segment at a time, such as shown in Figure 3. On the start of a trial, or when a participant clicked the *show outline* button, the outline would be indicated with a flashing red line around the edges of the segment for 1 second. On the right of the image was a vertical slider, ranging from 0 at the bottom with the label “not [attribute]” to 100 at the top with the label “[attribute].” On the right of the slider was a small box indicating the current value selected. Participants could move the slider using the mouse. On a left-click the participants could progress to the next trial. A button allowed the participant to go to the previous trial.

#### Exclusion criteria

As discussed above in the AMT section, we are capable of adding AMT *qualifications,* in an effort to improve data quality. First, we added three default *qualifications,* namely (1) that each participant needs to have completed at least 1000 tasks, (2) that each participant needed to have at least 95% of those tasks approved, and (3) that the participants were located within the United States of America.

Furthermore, we noticed in pilot experiments that some observers seemed to respond both quickly and randomly. Because their actual response cannot be an exclusion criterion (we cannot know what they perceive), we deemed it wise to use response time as selection criterion: if observers on average responded below one second, their data were excluded for further analysis.

#### Analysis

For the analysis of the data, we used several statistical methods and techniques. We will look into the intraobserver and interobserver correlations. Furthermore, we use principal component analysis (PCA) on the perceptual data. This technique applies an orthogonal transformation to data to produce a new set of uncorrelated variables, such as components. These components are ordered on the explained variance within the original data, where the first component explains the largest portion of the variance within the original data. Last, we also make use of a Procrustes analysis, which tries to find the best fit for a set unto a target set by minimizing the linear distance between points in the original set and the target set.

## Results

### Data quality; intracorrelations and intercorrelations

First, we analyzed the internal consistency by calculating the intra- and interobserver correlations. Each task contained three repetitions of each stimulus and was judged by 10 different participants for each material attribute. The average intraobserver correlation is 0.76 (*STD* = 0.08), which is higher than the average of 0.48 (*STD* = 0.16) for the interobserver correlation.

**Figure 4.**
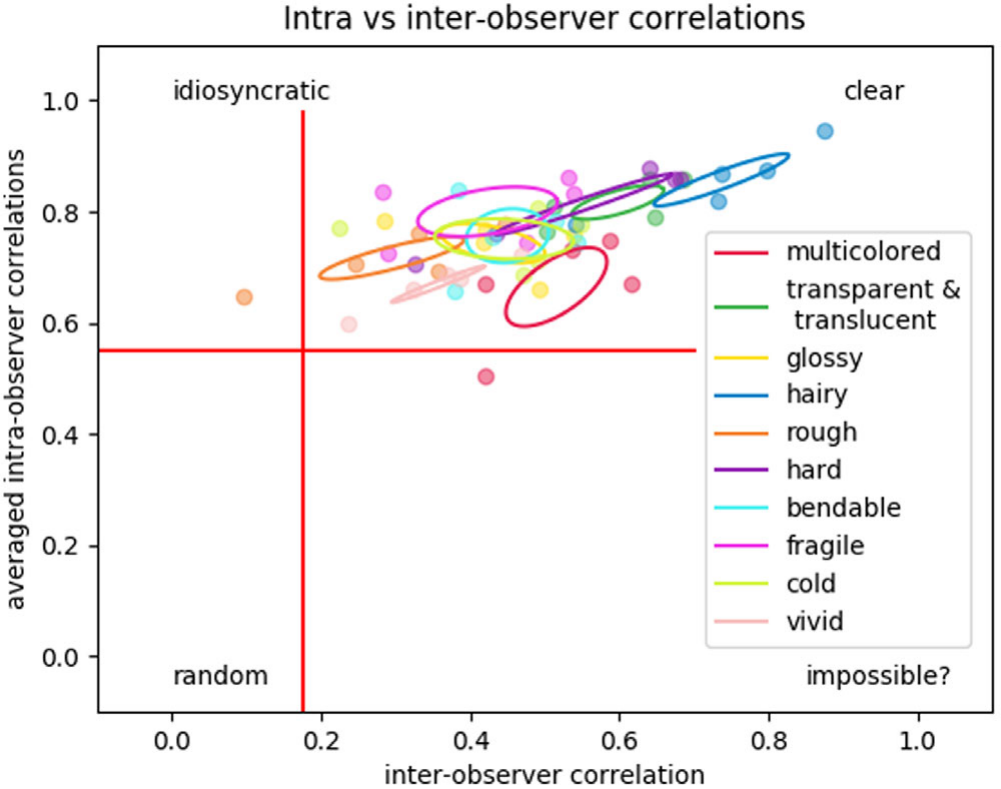
Each of the 50 set/attribute combinations expressed in a two-dimensional intraobserver/interobserver correlational space. The data are color-coded to indicate the material attribute that was judged. Ellipses (1 SD) are fitted for each material attribute based on the five experimental blocks relating to that attribute. The red lines represent the one-sided 5% alpha significance level, with 88° and 8° of freedom for intraobserver and interobserver correlations, respectively.

We plotted the correlations in Figure 4, where each point corresponds to one of the 50 set/attribute combinations, with the intraobserver correlation as a function of the interobserver correlation. Note that each of these 50 combinations was rated by a different group of 10 participants. We fitted an ellipse around the five points that belong to the same attribute. The distribution along the intraobserver axis shows that participants are, in general, consistent and that there is very little difference between the material attributes. The distribution of the interobserver correlations shows a larger spread, implying participants do not always agree among each other. The inverse, the small spread on the averaged intraobserver correlations indicate the high agreement rate within participants. Additionally, the material attributes cluster together, but the clusters are spread out over the interobserver correlation dimension implying that the magnitude of (dis)agreement between participants is material attribute dependent.

### Material judgments

**Figure 5.**
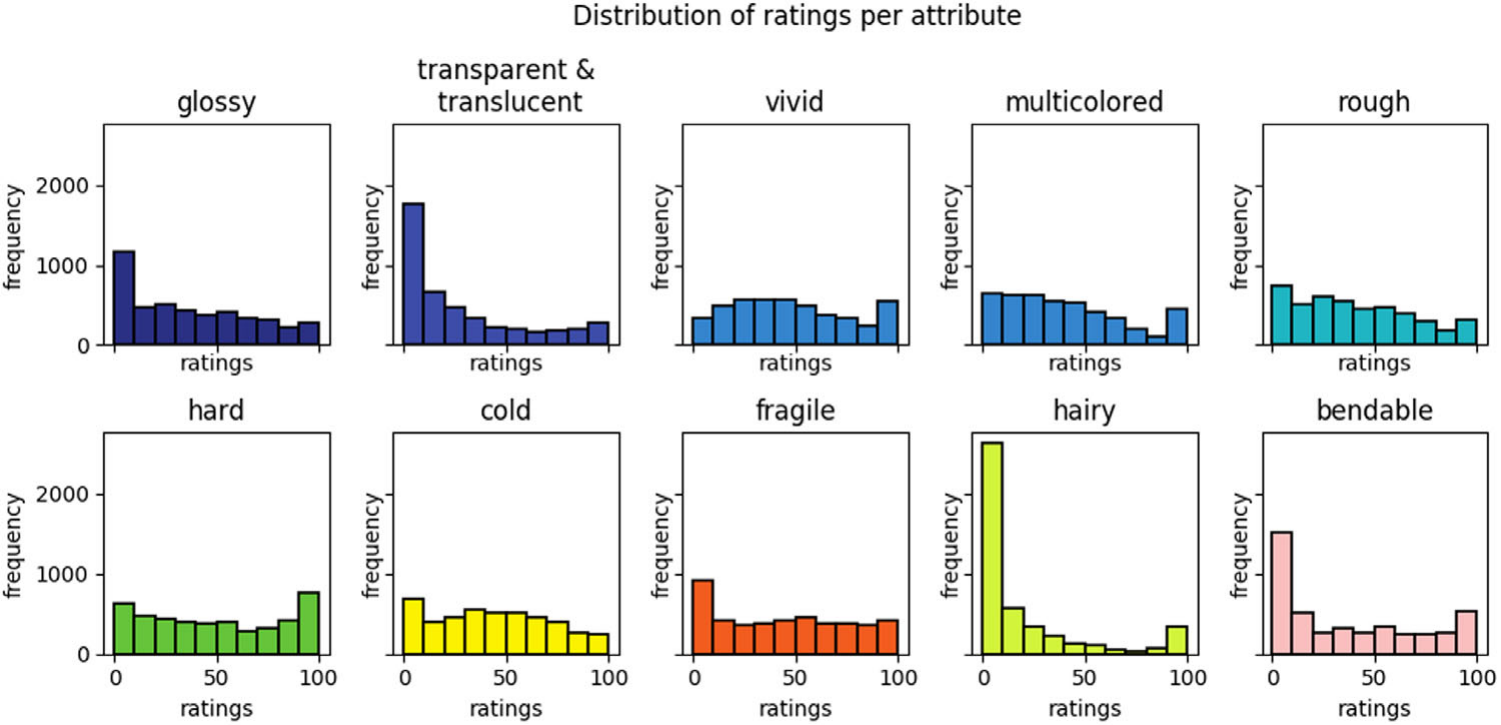
Distribution of all the judgments per attribute for all materials. The colors are in reference to the colors used by Fleming et al. (2013).

We collected a total of 135,000 human judgments about how much a specific stimulus depicted a specific attribute. We have plotted the distributions of these ratings per attribute in Figure 5. At a glance, it becomes clear that the distributions are generally broad and flat, except for some attributes at zero. The stimuli cover the whole range for each attribute, and when an attribute is present it is more or less equally likely to be present in any quantity.

**Figure 6.**
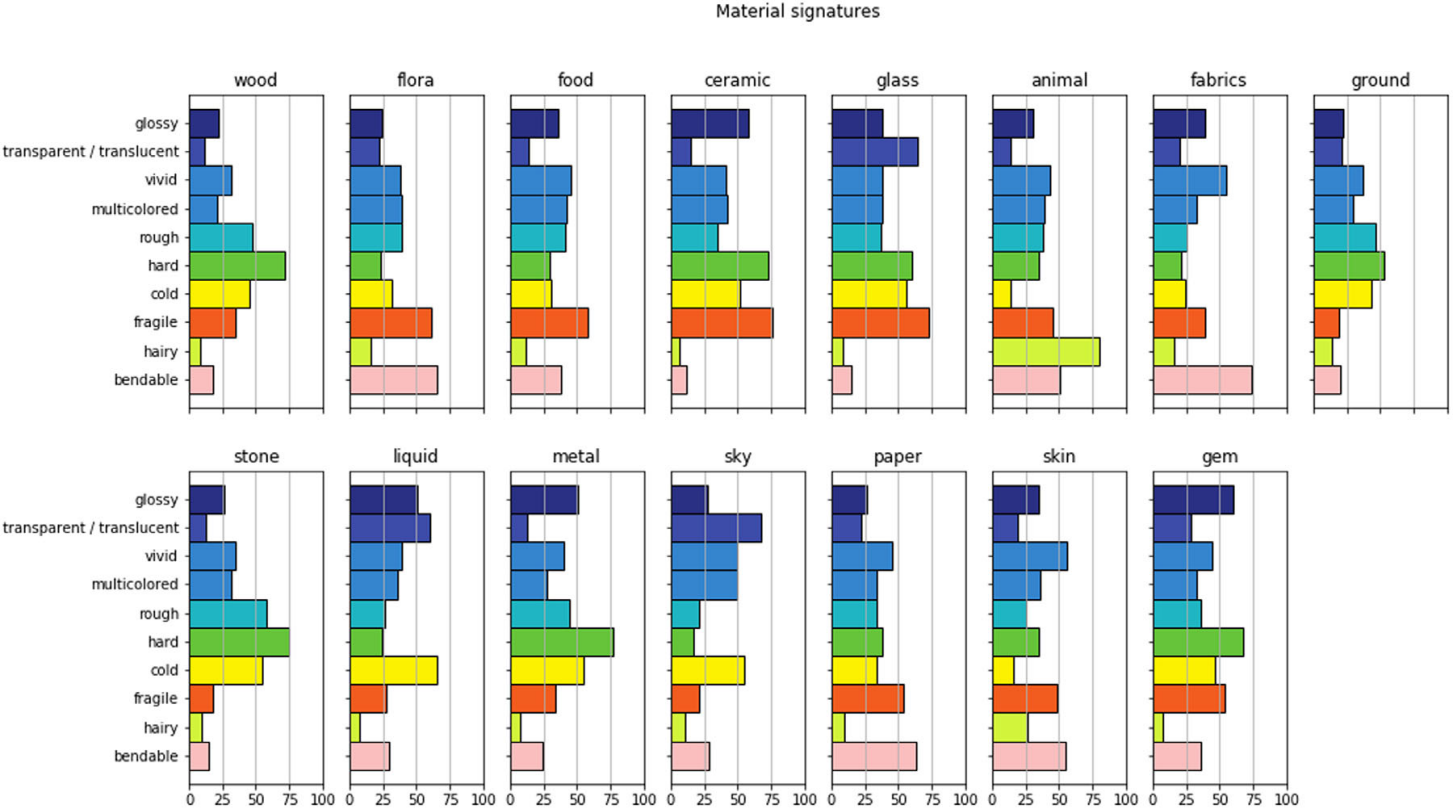
The averaged ratings for each attribute per material.

**Figure 7.**
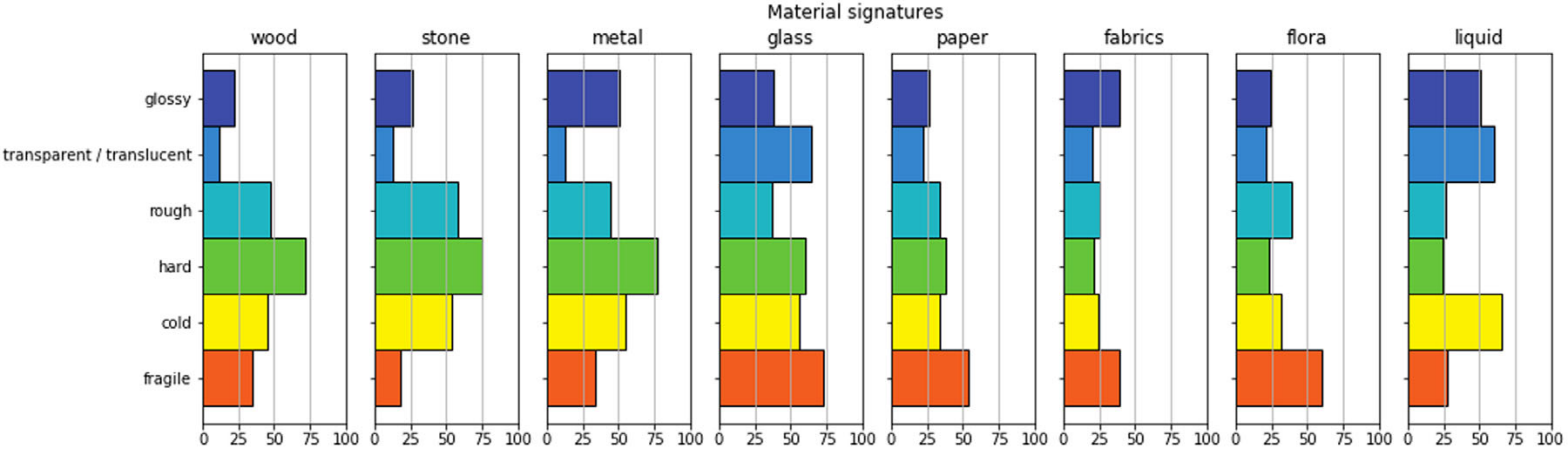
A recreation of the average rating for the attributes and materials from Fleming et al (2013), for the materials and attributes that are shared between our study and Fleming's study. Note that in our study, we split up colorfulness into vivid and multicoloredness. Figure adapted with permission; original copyright belongs to ARVO.

We visualized the averaged distributions of material attributes for each material in Figure 6. Here, we found some remarkable similarities with those reported by Fleming et al. (2013), and therefore we reproduced these in Figure 7. To make an accurate comparison, it should be noted again that our study did not use the same set of attributes as did Fleming et al. (2013). Our signature included *hairy* and *bendable,* while excluding *naturalness* and *prettiness.* Furthermore, we split *Colorfulness* into *vividness* and *multicoloredness* and included *translucency* into *transparency.* What we observe is that the distributions seem to follow the same pattern for the materials that are in common between our study and Fleming et al. (2013). To quantify this relationship, we performed a non-parametric Wilcoxon signed-rank test in which we paired the mean values for the materials and attributes that the current study has in common with the study of Fleming et al. (2013). Note that we equate Fleming's *transparency* with our “ *transparent/translucent*” and Fleming's *colorfulness* with our *vivid* and *multicolored.* The test showed that there was no significant difference between the attribute ratings for photographs and paintings (*Z*[56] = 790, *p* = 0.94).

### Material attribute correlations

**Figure 8.**
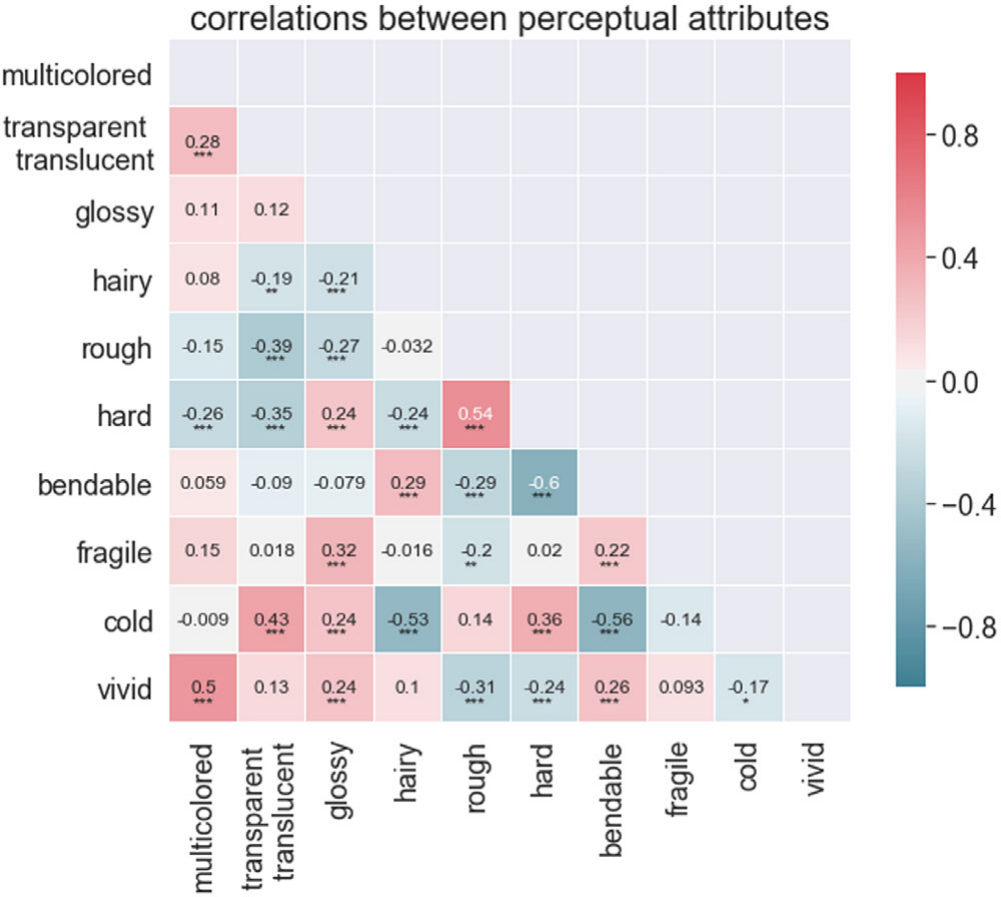
Correlation matrix heatmap, we have masked the values along the diagonal, which would always simply be 1 and the symmetrically identical values. * indicates *p* < 0.001, ** indicates *p* < 0.0001, and *** indicates *p* < 0.00001.

Correlations likely exist between the material attributes: a change in one attribute could lead to a predictable change in other attributes. We quantify these relations by calculating the correlations using Bonferroni adjusted alpha levels of .001, .0001, and 0.00001 (0.05/45, 0.005/45, and 0.005/45, respectively). These correlations have been visualized in Figure 8. The highest correlations are found between *roughness* and *hardness (r* = 0.54, *p* < 0.0001), and between *vividness* and *multicoloredness* (*r* = 0.5, *p* < 0.0001). The lowest correlation is found between *hardness* and *bendableness* (*r* = −0.6, *p* < 0.0001). The majority—33 of 45—of the attributes pairs only displayed a small (i.e., *r* < 0.3) correlation. This implies that although there is overlap, most attributes cover a distinct area of a high-level material-feature space.

### PCA

**Figure 9.**
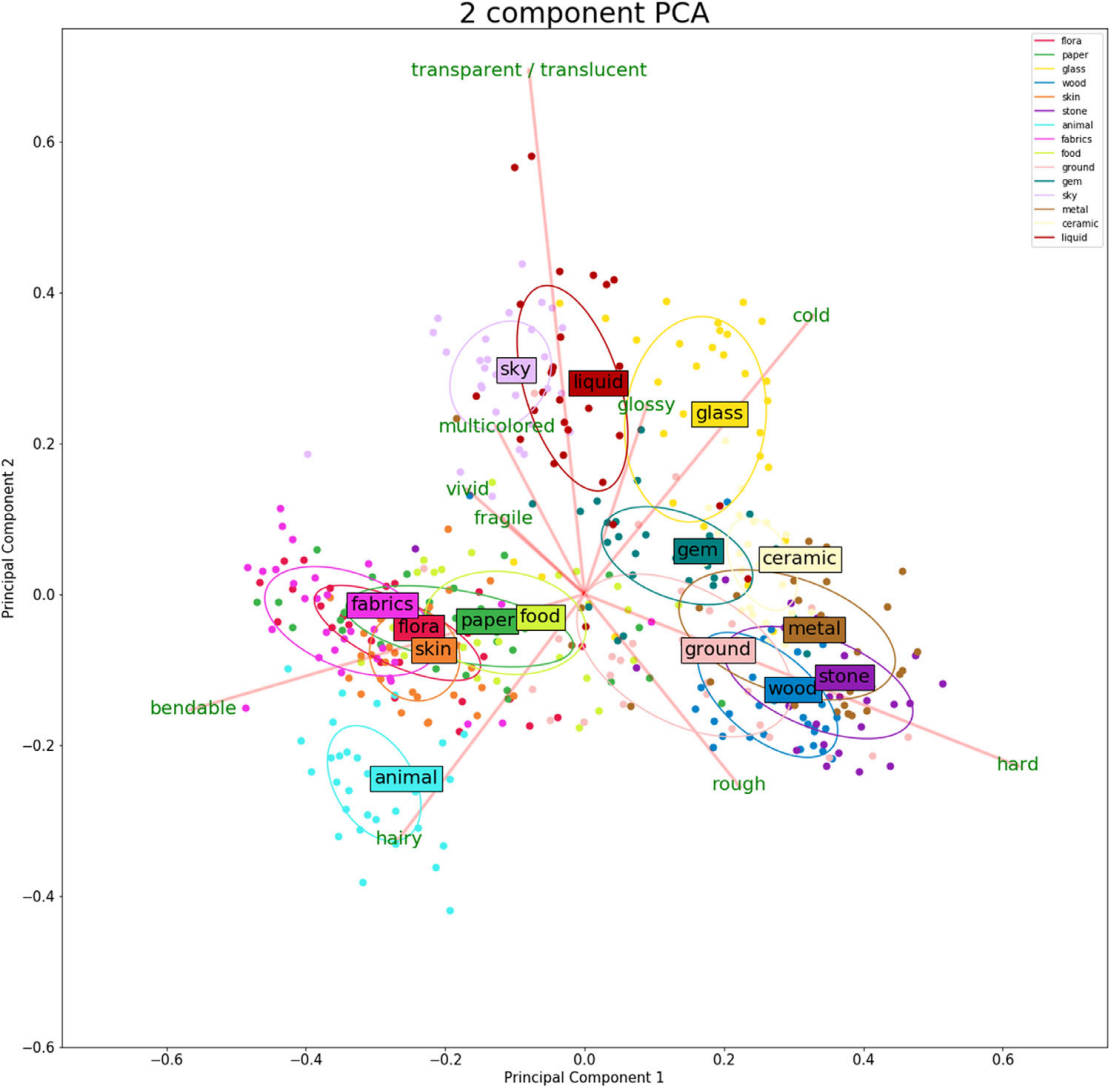
A visualization of the first two PCA dimensions. The color of the points relates to material class identity. The factor loadings of the original dimensions are plotted as red vectors. Lastly, we fitted ellipsoids (sd = 1) for each material class. Note that the PCA is not fed any class data; the clustering of material classes observed is thus purely based on the perceptual data.

**Figure 10.**
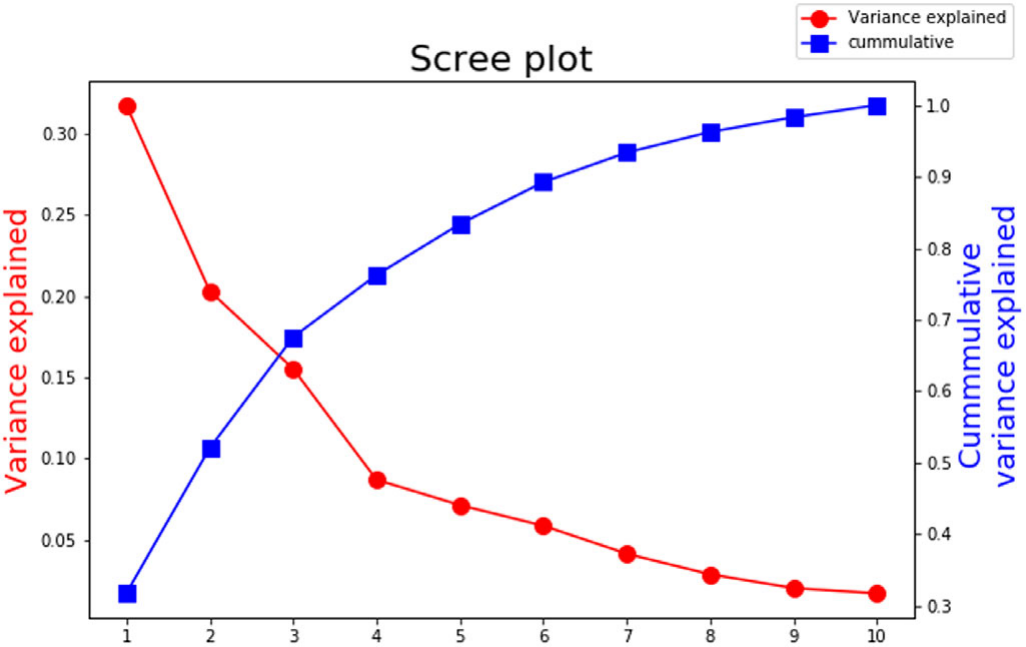
Scree plot for the PCA visualized in Figure 9.

To analyze the relationship between material attributes and to determine whether material attributes can predict material class identity, we applied a PCA to uncover the underlying multidimensional attribute feature space. This technique applies an orthogonal transformation to remap the original data set in such a way that the new dimensions (components) are linearly uncorrelated, and ordered by the quantity of variance, where the first dimension explains the most variability within the original dataset. We have visualized the first two components in Figure 9, which explain 52% of the variability within the data. Adding a third, fourth, or fifth component captures 68%, 76%, and 83% of the variability, respectively. These numbers are roughly comparable to the two numbers Fleming et al. (2013) reports: 62% for the first two PCs and 93% for the first five PCs. However, it should be noted that our measured dimensions are not identical (see *Attributes and image statistics* in the method section). We have plotted a full scree plot in Figure 10 and added the factor loadings for the first four components in Table 1.

**Table 1.** Factor loadings for the first four principal components.

	PC1	PC2	PC3	PC4
Multicolored	−0.126	0.221	0.07	0.511
Transparent/translucent	−0.08	0.693	−0.2	0.019
Glossy	0.09	0.25	0.471	0.157
Hairy	−0.267	−0.325	−0.063	0.583
Rough	0.221	−0.253	−0.078	0.023
Hard	0.621	−0.227	0.305	0.152
Bendable	−0.562	−0.153	0.171	−0.341
Fragile	−0.116	0.1	0.757	−0.167
Cold	0.326	0.368	−0.093	−0.088
Vivid	−0.167	0.138	0.141	0.445

**Figure 11.**
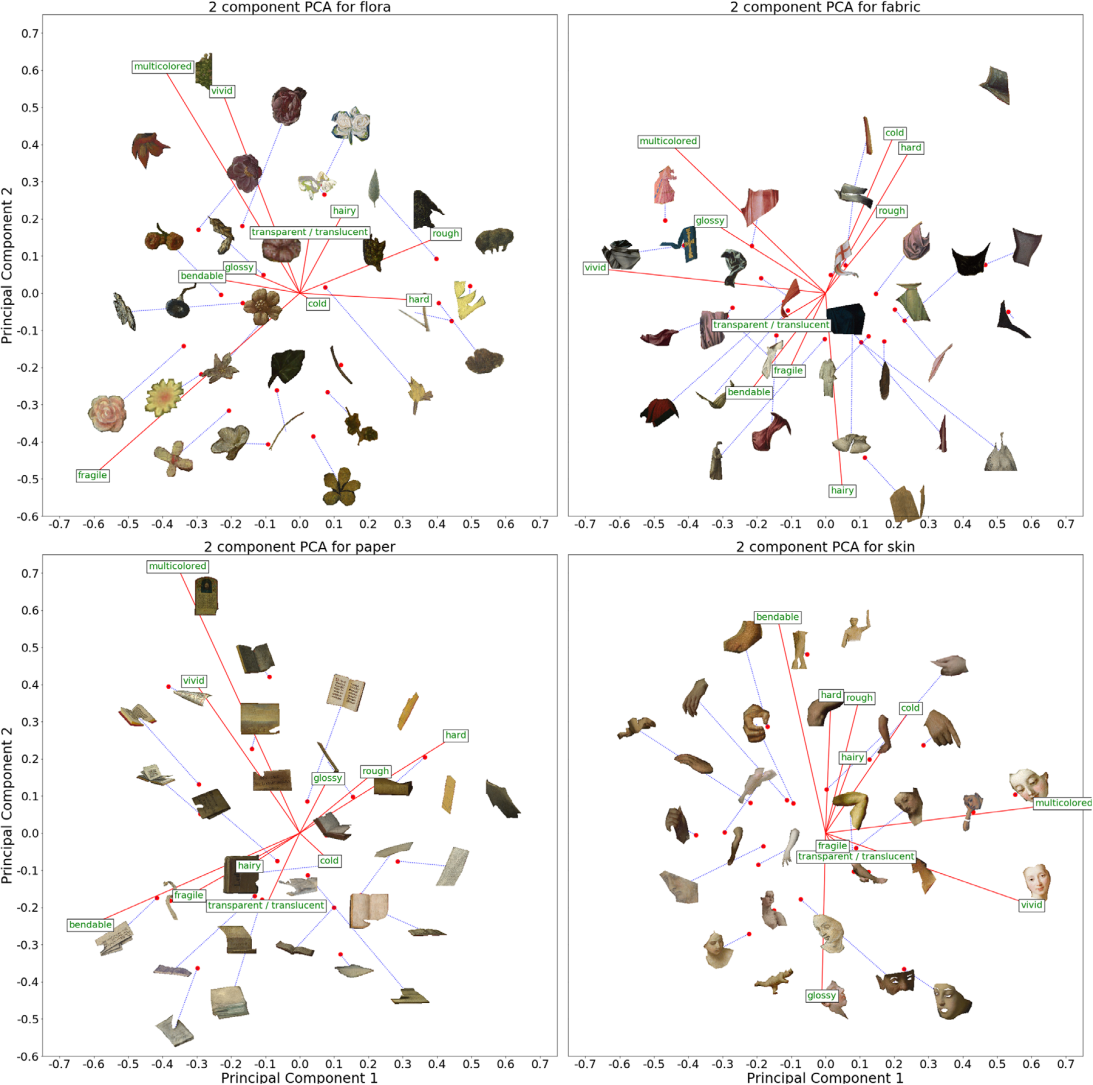
Four visualizations of the first two primary components for the material-specific PCA for flora, fabric, paper, and skin. Each PCA was run with only the 30 stimuli per material. The red vectors indicate the factor loadings of each attribute. We plotted the actual stimuli within the PCA space. The blue lines connect the stimuli to their actual position within the space when the stimuli would otherwise overlap. The ellipse is fitted around the points (1 SD).

We also ran a PCA for each material, that is, with only the 30 datapoints belonging to that specific material, as opposed to all 450 datapoints for all materials. We visualized these for *paper, skin, flora,* and *fabric* in Figure 11. The remaining material plots are included in the supplementary materials.

We have included all the factor loadings for all the PCAs (1 × global and 15 × material specific) within the supplementary materials. Next, we applied a Procrustes analysis to map each material-specific PCA onto the associated data-points within the global PCA space, that is, the 30 segments for one material-specific PCA were mapped onto the 30 corresponding segments within the global PCA. Here, the residual error quantifies how much a material-specific PCA deviates from the global PCA. Or, inversely, how similar the variance within one material is in comparison to the global variance found between all materials. We applied the Procrustes analysis on the first two components, as opposed to all ten. The reason for this is simple: a PCA works by applying an optimized transformation on a data set, whereas the Procrustes analysis tries to find an optimized transformation to map one dataset onto another. Consider that the material-specific PCA dataset is a subset of the global PCA dataset. This means the raw data for the PCAs are the same but have undergone different transformations within a 10-dimensional space.

Thus, applying a 10-dimensional Procrustes analysis would perfectly map the material-specific subset onto the global PCA leaving a residual of exactly 0. Instead, we take the two primary components that explain the major part of the variability. Note that the loadings of the first two PCA dimensions can change from the global to the material-specific models and that materials with a larger variability can have a larger influence on the global variability relative to materials with less variability. The residuals of the Procrustes analysis are listed in Table 2. We also used randomly generated data points drawn from a uniform distribution and mapped these to each of the material subsets within the global PCA using the Procrustes analysis. We repeated these 10,000 times, for each material, to find an averaged residual error of 0.9508 which functions as a comparison. The results are visualized and ordered in Table 2 and show that the residuals range from 0.14 to 0.74 and are all smaller than for the random set. This shows that intramaterial variations are described relatively well by the variation in the global PCA space, but for some materials better than others.

**Table 2.** Table of residuals of the Procrustes analysis.

Material	Residual
Fabric	0.14
Metal	0.19
Stone	0.22
Ground	0.23
Glass	0.25
Food	0.28
Paper	0.36
Wood	0.36
Liquid	0.37
Ceramic	0.37
Flora	0.5
Animal	0.46
Sky	0.53
Gem	0.72
Skin	0.74
Random	0.95

Lower residuals indicate more generic materials.

### Image statistics

**Figure 12.**
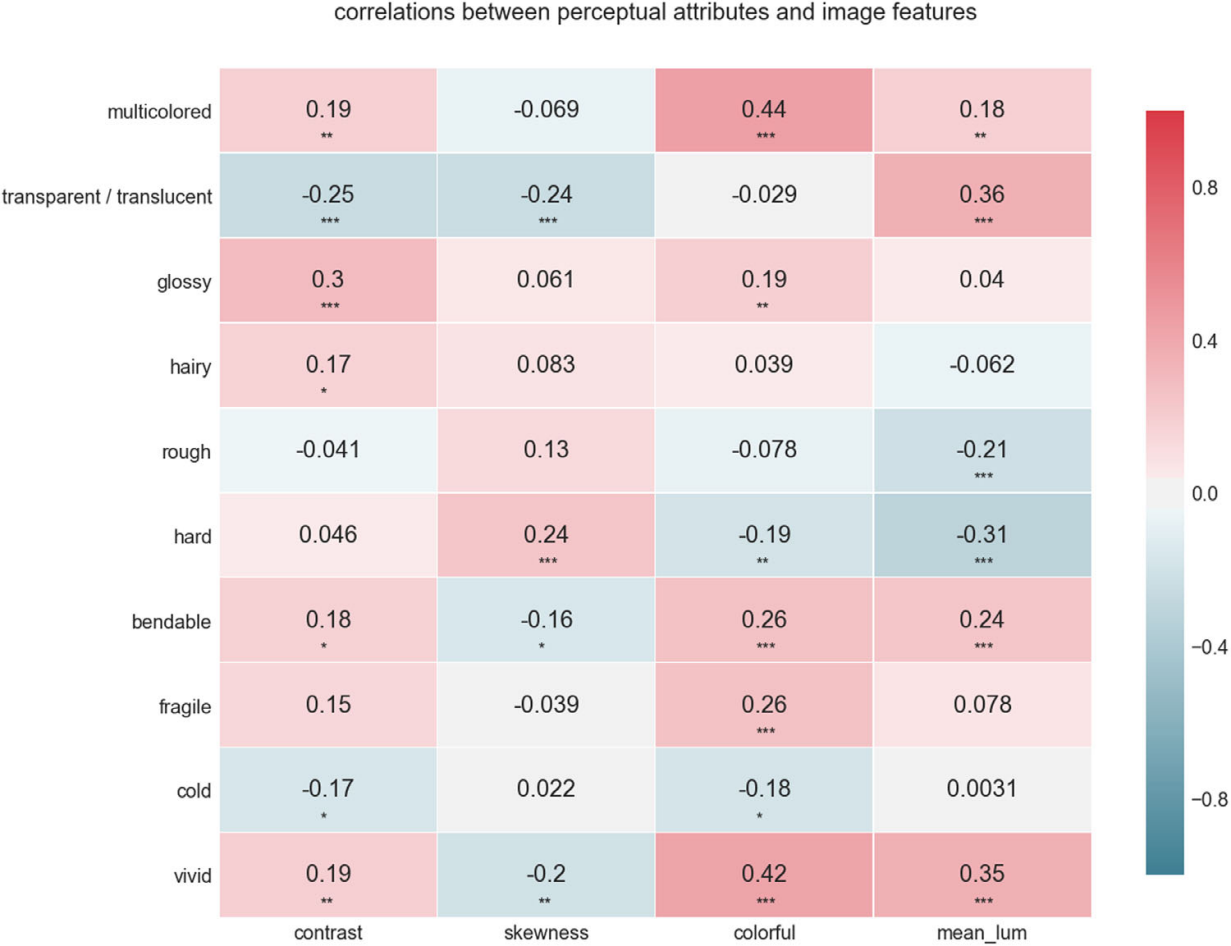
Correlation between the perceived perceptual attributes and image statistics (i.e., image statistics). * indicates *p* < 0.0013, ** indicates *p* < 0.00013, and *** indicates *p* < 0.000013.

As detailed in the Methods section, we calculated simple histogram-based image statistics for each image stimuli. We correlated the material attributes with these image statistics, both averaged over materials and per material. We adjusted the alpha levels using Bonferroni correction to .0013, .00013 and .000013 (.05, .005, and .0005 divided by 40 respectively). Over all materials generalized, we found some correlations. *Colorful* correlated with *multicolored* (*r* = 0.44, *p* < 0.00013) and with *vivid* (*r* = 0.42, *p* < 0.00013), suggesting our color metric indeed captures *multicoloredness* to a certain degree. Furthermore, *mean luminance* correlated with *transparent/translucent* (*r* = 0.36, *p* < 0.00013) and with *hardness* (*r* = −0.31, *p* < 0.00013). These correlations and the remaining, smaller correlations have been visualized in Figure 12. The significant correlations per material have been listed in Table 3. Here the colorful-multicolored and colorful-vivid relationships are often found to be significant. In addition, the skewness of the luminance distribution and mean luminance are found to be related to specific attributes in a material-dependent manner.

**Table 3.** Significant correlations between perceived material attributes and image statistics.

Material	Perceptual attribute	Image statistic	*r*	sig
Ceramic	Multicolored	Colorful	0.75	***
Glass	Vivid	Mean luminance	0.6	*
Gem	Multicolored	Colorful	0.75	***
	Bendable	Skewness of the luminance distribution	−0.81	***
	Bendable	Mean luminance	0.83	***
	Fragile	Skewness of the luminance distribution	−0.73	***
	Fragile	Mean luminance	0.72	***
Sky	Vivid	Colorful	0.69	**
Fabric	Transparent/translucent	Skewness of the luminance distribution	−0.58	*
Wood	Multicolored	Colorful	0.73	***
	Vivid	Colorful	0.63	*
	Multicolored	Mean luminance	0.68	*
Metal	Multicolored	Colorful	0.57	*
Ground	Multicolored	Colorful	0.68	**
	Vivid	Colorful	0.58	*

* indicates *p* < 0.0013, ** indicates *p* < 0.00013, and *** indicates *p* < 0.000013.

## Discussion

In this study, we collected human perceptual judgments for 10 material attributes for paintings of 15 material classes. The consistency within participants indicates that participants understand and are capable of performing the task, which shows that our experimental setup allows for measuring the perception of material attributes in paintings via AMT and supports the validity of the data, whereas the inconsistency between participants shows that individual differences exist in how participants interpret the depictions. Additionally, we found that the material signatures and the material feature PCA spaces show many similarities to those of Fleming et al. (2013) based on material photographs, as well as of Zhang, de ridder, Barla, & Pont (2019) based on mixtures of canonical reflectance modes, which implies that the perception of material properties functions independently of the medium of depiction and the structure found represents generic key components underlying material perception. Lastly, we looked at the residuals that result from mapping material-specific PCA data onto the global material PCA and found that the variation within materials is partially explained by the variation between materials, but that this varies depending on the material. Below we will discuss these findings in more detail.

In the task, participants were shown a square bounding box and asked to use only the segment, outlined in red (see Figure 3) when they make their judgments. Participants may make their judgments based on the object category inferred from this red outline. However, it should be noted that for the vast majority of segments, the materials are partially occluded by other materials or objects and tend not to be informative of the object identity, see, for example, those in Figures 1 and 3.

Besides being a measure of the internal validity, the high consistency displayed by participants shows that the perception of material attributes is distinct and that participants have a clear perception of these attributes. Despite this clear perception, disagreement between participants does exist. The magnitude of this disagreement—which ranged from 0.01 to 0.87—appears to depend on the perceptual attribute. Roughness induced the highest level of idiosyncrasy, whereas hairiness is the most consistent between—and also within—participants. The overall pattern of (in)consistencies between participants for the perceptual attributes in our results appears to be very similar to those reported by Fleming et al. (2013); however, it is interesting to see that roughness is one of the most consistent in their results, whereas in our study it is the least consistent between participants. It is unclear why these results differ. Possibly, roughness is too multidimensional to be measured in one scalar measure; even for a single type of surface structure it was found that its roughness perception was multidimensional (Padilla, Drbohlav, Green, Spence, & Chantler, 2008).

In the experiment conducted by Fleming et al. (2013) they found that materials tend to display statistical regularities, such as glass tending to look glossy, transparent, smooth, hard and so on, while water also tends to look glossy and transparent, but not at all hard. He postulated that these distinctive features can be interpreted as a *signature* of a material class. In this study, we found that the material signatures for painted materials are also distinct and that some materials appear to be more similar to each other than others. For example, wood and stone have a very similar material signature, and glass and liquid are almost identical except that glass is—obviously—harder and more fragile. Also, many of the between-attribute correlations seem intuitive, such as the negative correlation between hardness and bendableness, as well as the negative correlation between hairy and cold. Furthermore, we find some remarkable similarities with the material signatures reported by Fleming et al. (2013), which shows that the perception of photographed and painted materials results in similar associations, which suggests a generic underlying mechanism.

When looking at the first two components of the PCA we find that materials tend to cluster together, but that clusters for different materials can overlap. This implies that the perceptual judgments are material specific in terms of perceptual attributes, but that extracting a specific material identity based solely on the perceptual attributes measured in this study would likely be prone to errors. Possibly by adding a more extensive list of perceptual attributes, a predictor model could predict the material class identity. Furthermore, when looking at the PCA visualization, it is again interesting to note the similarity between the data presented here and the PCA dimensions reported by Fleming et al. (2013). This implies that material perception functions independently of the medium of depiction.

One could argue that this can be explained by semantic knowledge: material classification is extremely fast, and after classification we gain access to semantic information, which in turn could have a top-down influence on the perception of perceptual attributes (Xiao, Sharan, Rosenholtz, & Adelson, 2011; Wiebel et al., 2013; Sharan, Rosenholtz, & Adelson, 2014). Then, the estimation and perception of material properties could be argued to be driven by a top-down influence from material recognition. This top-down influence would then also be independent of the medium. To test this idea, Fleming et al. (2013) conducted a second experiment, where participants rated the material attributes of semantic stimuli (i.e., only material class names). They found that material property ratings for the semantic-only represented material classes were very close to the cluster centers for photographic representations. It would be naïve to claim that semantic top-down influences can fully explain material perception since we are capable of making judgments based on material properties within a material class (which fruit looks fresher? which sweater looks softer?). It does, however, imply that our perception might be influenced by semantic top-down influences when viewing materials. Furthermore, Zhang et al. (2019) had participants perform a material probing task on a canonical set of computer rendered base images, where material perception could only rely on material reflectance since there was no semantic information. They found a PCA space that is similar to our PCA space and the one reported by Fleming et al. (2013). Thus, whereas semantic information might explain a portion of material perception, it does not explain the perception of intraclass variations. Thus, although semantic information might explain a portion of material perception, it does not explain the perception of intra-class variations. Furthermore, because the global PCA structure cannot entirely be explained by semantic information, it is implied that a portion of the global PCA structure (i.e., the portion not explained by semantic information) is independent of the medium of depiction.

The PCA space visualizes the majority of the perceptual variability of the materials and in doing so, shows how materials are—and importantly, how they are not—related. We were interested in seeing how similar the variability within one material is in comparison to the variability found between all materials. To do so, we took the variability within one material and analyzed how well this mapped unto the variability between all materials. To quantify, we performed the Procrustes analysis. Here, the lower the residual error is for a material, the closer the variability within the material resembles the variability between materials. The first, intuitive result is that different materials vary differently across the perceptual attributes we measured. This effect could be highly dependent on the stimulus set. However, if we consider the previous results, namely that different stimulus sets have remarkably similar PCA spaces, even with different methods of depictions (e.g., paintings in our study, photographs in Fleming et al. [2013] and reflectance modes mixtures in Zhang et al. [2019]) and that the material signatures are very similar for photographic and painted images (see Figures 9 and 10). Furthermore, the finding that different materials varied differently across perceptual attributes further suggests that the similarities we find are not just a semantic effect. If it was merely a semantic effect, it would be more likely that the Procrustes residuals would show little variability between materials. Looking at specific materials, the residuals showed that fabric, metal, and stone are relatively generic materials: the variability within these closely resembled the variability between all materials. Gem and skin were found to be much more distinctive materials, because the variability did not resemble the global variability. In summary, the residuals of the Procrustes showed that different materials varied differently across perceptual attributes. Some intramaterial variations are quite generic; that is, they closely resembled the global material PCA space. However, other materials are more unique and resembled the global variability.

It has previously been proposed that variations in the perception of specific material attributes could be explained by image statistics (Motoyoshi et al., 2007; Baumgartner & Gegenfurtner 2016), but this has also been debated (e.g., Kim & Anderson, 2009). Considering the large amounts of data collected in our study, we decided to calculate several simple, histogram-based image statistics, to see whether those could explain variations of the perceptual attributes. It appeared that the small set of image statistics we used did not correlate strongly with the perceptual attributes across all materials. This could perhaps be expected, after finding that the Procrustes residuals varied across materials. We did find some weak and moderate correlations, however, and the correlation we found between transparency/translucency and the mean luminance of the stimuli seems an interesting finding. It has previously been argued that the average luminance is a poor predictor for transparency in natural images and that this is due to the luminance of an object being strongly influenced by scene illumination and the objects spatial and directional properties, shape, and context (Koenderink & van Doorn, 2001; Fleming & Bülthoff, 2005; Fleming et al., 2011; Fleming, Jensen & Bülthoff, 2004). When looking at the correlations per material, it is interesting to note that the majority of the correlations, as well as the strongest, were all found for gem. Perhaps, it is possible that this material simply shows stronger optical effects than other materials.

As previously noted, the image database we used is different from existing image sets, such as the FMD, because each image comes from a certain artist and a certain period. Although the sample size for the perception experiment is relatively small with respect to all paintings at our disposal, it does give us some idea of interesting future directions for the study of art and perception. For example, we conducted a small pilot, not reported here, where we found that gems are perceived as glossier for recent paintings relative to older paintings. A typical art historical hypothesis would include the invention of oil paint that supposedly increased the convincingness of materials. Yet, most of our paintings are after this invention and the material rendering revolution that van Eyck caused in the fifteenth century. But there can be many other reasons and possibly even patterns that have not yet been identified in art history. With our continued work on creating the painting database of material depictions we hope to further investigate these questions.

## Gallery URLs

• https://www.nationalgallery.org.uk
• https://www.rijksmuseum.nl/en
• https://www.museodelprado.es/en
• https://www.nga.gov/
• https://www.nationalmuseum.se/en/
• https://www.getty.edu/museum/
• https://www.metmuseum.org/

## Supplementary Material

Supplement 1

Supplement 2
