# Supplementary material for: Painterly depiction of material properties
Source: J Vis. 2020 Jul 7;20(7):7. doi: 10.1167/jov.20.7.7 (PMC7426625; doi:10.1167/jov.20.7.7)
Supplement: Supplement 2 [file jovi-20-7-7_s002.docx]

# Supplementary materials.

Here we include a short description of the Amazon Mechanical Turk platform. We follow this by methodological details related to the gathering of the data that were not included in the main text. Furthermore, we have added here the material-specific PCA visualizations that were not included in the main paper. Next, we list the factor loadings of the two first principal components of all the PCAs that we ran, for all material specific PCAs and the global PCA in the bottom, which contained all materials. A list of all the paintings used in the perceptual experiment can be found in the separate .xlsx file.

## Amazon Mechanical Turk

For all data collection, we use the online labor marketplace Amazon Mechanical Turk (AMT), colloquially referred to as MTurk. On this platform, people can sign up as workers, or turkers, to perform micro-tasks for requesters. The platform has been popular for companies that require simple human-intelligence tasks such as transcriptions. In recent years, the MTurk platform has also seen increased use from the social sciences (Peer, Vosgerau & Acquisiti, 2014; Bohannon, 2016) and computer sciences (Bell, Upchurch, Snavely, & Bala, 2013, 2015; Papadopoulos, Uijlings, Keller, & Ferrari, 2017; Su, Deng, & Fei-Fei, 2012).

              Workers are free to choose what tasks to perform and have the option to stop at any moment if they so desire. They are paid per task, where each task can last a few minutes (the usual) up to multiple hours. The requester is given a chance to review the submitted work and has the option to either accept or reject the work based on the quality. Workers are not paid for any rejected work. For the experiments reported below we accepted all work as it is clearly impossible to define quality in our case: there is no right or wrong perception.

              Workers can be selected *before* the task. This can be achieved by using qualifications that are based on demographic criteria such as geography and age, or reputational criteria such as requiring that a worker has completed a minimum number of tasks with a certain percentage of acceptance. For example, it is common – as these are the default values on AMT – to require that a worker has completed at least 1000 tasks, of which at least 95% percent has been accepted. With these qualifications, workers can be restricted from starting tasks.

### Step 1: Collecting Paintings.

We downloaded 17936 images from the digitized open-access painting collections from seven galleries. The galleries are listed in table 1. Most of these images were collected via web scraping the websites of these seven galleries. Web scraping was done with Python, making use of the Beautifulsoup, Selenium, and urllib modules. For the Rijksmuseum, we used their API to directly download each image individually. For the National Gallery of London, we used peer-to-peer downloading to download the collection.  The URLs for each gallery have been supplied in the references.

              For each image, we also obtained the original meta-data from the gallery, which contains for example information about the title of the artwork, the artist, and an estimated creation date.

              Next, we filtered out 661 monochrome images using a linear regression on the 3D color data keeping a total of 17275 paintings. We have not applied any color management or color corrections techniques, nor do we have any information into what color management the respective galleries applied.

| **Gallery** | **Count** |  | **Country** |
| --- | --- | --- | --- |
| Rijksmuseum | 4657 |  | Netherlands |
| NationalMuseum | 2924 |  | Sweden |
| The Metropolitan | 2729 |  | USA |
| National Gallery of London | 2402 |  | United Kingdom |
| National Gallery of Art | 2132 |  | USA |
| Museo Nacional Del Prado | 2032 |  | Spain |
| Getty | 399 |  | USA |

Table 1. Each gallery and the number of images we downloaded from their digitized open-access gallery.

### Step 2: Collecting materials

To identify what materials are perceived to be depicted in the collected paintings 1571 AMT participants completed a total of 36838 tasks. The majority *(n=*511*)* of participants only completed the task once, while some participants completed a much larger number of tasks: two participants completed more than 1000 tasks each and an additional 6 participants completed over 500 tasks each*.* Each task paid 0.02 USD and presented participants with 40 paintings and a target material. An example of this can be seen in figure 1, where the target material is *fabric.*  Participants could scroll through the webpage to view all the paintings. The target material would be one of the materials listed in the *Materials and Attributes* section. Participants were instructed to use the mouse to click each painting where they perceived the target material to be present. If a painting was clicked the painting would gain an orange outline and a small textbox in the bottom right corner would change from “no [material]” to *“*[material]*”.*
 Before starting the task, participants would be presented with a short tutorial. In the tutorial, the task was explained followed by a short description of the specific material was given. At the end of the tutorial, participants had to complete a small number of test trials. The number of test trials varied per material, between 3 to 5. In each test trial, the participants were shown one painting at a time and had to click the correct button, indicating whether the painting did or did not depict the requested material for that trial. Upon giving a wrong answer, a participant was immediately given feedback and asked to redo the same trial. Once all test trials were completed, the real task would commence. Once a tutorial was completed for a specific material, the participant would not be shown the tutorial for that material again
 For each painting/material combination, we collected responses from at least 5 participants. If at 80% or more of these participants responded that a painting depicted a material, we considered that painting to depict that material for the continuation of this project.

### Step 3: Collecting segmentations

In the next step, AMT participants would be tasked with segmenting the target material, where the target material was one of the materials that is depicted in the painting, as indicated in step 2. Participants were told to “find the largest area displaying [material] and trace its outline as accurately as you can”, where [*material*] would be replaced by one of the materials listed in the *Materials and Attributes* section. A screenshot of the task can be seen in figure 2.
 To make a segment, a participant could click anywhere on the presented painting, which would create a point on the painting. Consecutively placed clicks would be connected with a blue line, and a line would continuously be drawn between the last placed point and the mouse. Once the outline of the chosen material instance was traced, the last point could be connected to the first point by hitting the *enter* key*,* a right-mouse click, or by pressing the *Close* button. Once *closed,* a participant could enter the *adjust mode,* either pressing the *Adjust* button or the *a* key. In the *adjust mode* individual points could be moved using *click-and-drag.* Additionally, participants could zoom in or out by scrolling, could move the painting using the arrow keys, or undo and redo the last action using the buttons or by pressing *ctrl-z* or *ctrl-y* respectively. Once participants were satisfied with a segment, they could finish the task by pressing the *submit* button.
 Initially, we placed no restrictions on which participants could perform the segmentation task. However, after we collected around 1700 segments from a total of 52 participants, we selected the five participants whom we judged to be the best, based on the observed quality of the created segments. From this point onwards, only these five selected participants were allowed to perform the task. This restriction increased the average number of points, i.e. vertices in the polygonal segmentation by 62% from 37 to 65, indicating that the average detail per segment increased. Additionally, the acceptance rate, i.e. the number of submitted segments that passed the quality control in the next step, improved by 20% from 0.58 to 0.7. The five selected participants completed 283, 455, 777, 1235 and 3082 tasks. In total, all participants together completed 7110 tasks, for 0.15 USD per task.


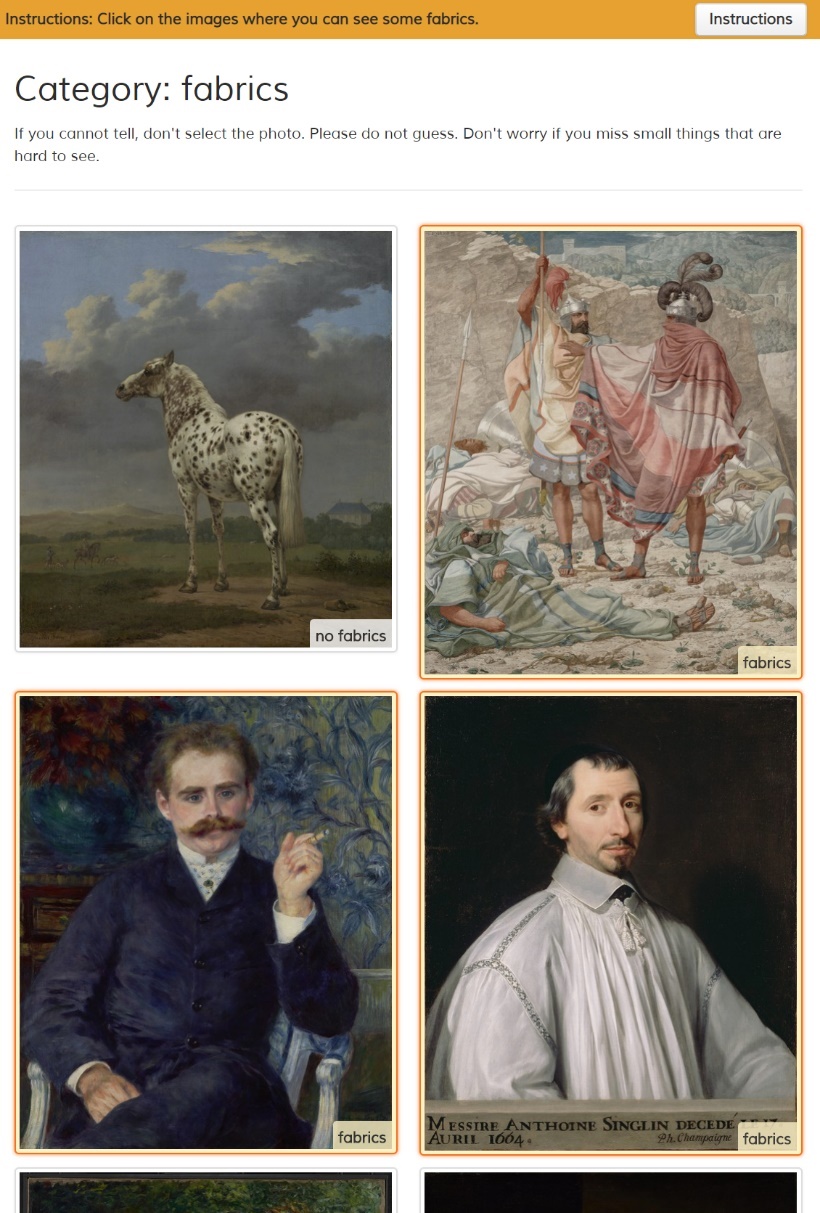


Figure 1. Example of the material-collection web task. After a tutorial, participants would see this screen. The yellow top bar shows the instructions in short, and the right-wards button opens a pop-up menu with additional instructions when clicked. The target material is indicated semantically. Participants would use the mouse to scroll through a total of 40 images. Once a painting was indicated (clicked) to contain the target material, the white outline (as can be seen in the top-left painting) changed to orange (as in the other 3 visible painting) and the label in the bottom right of the painting would change accordingly.


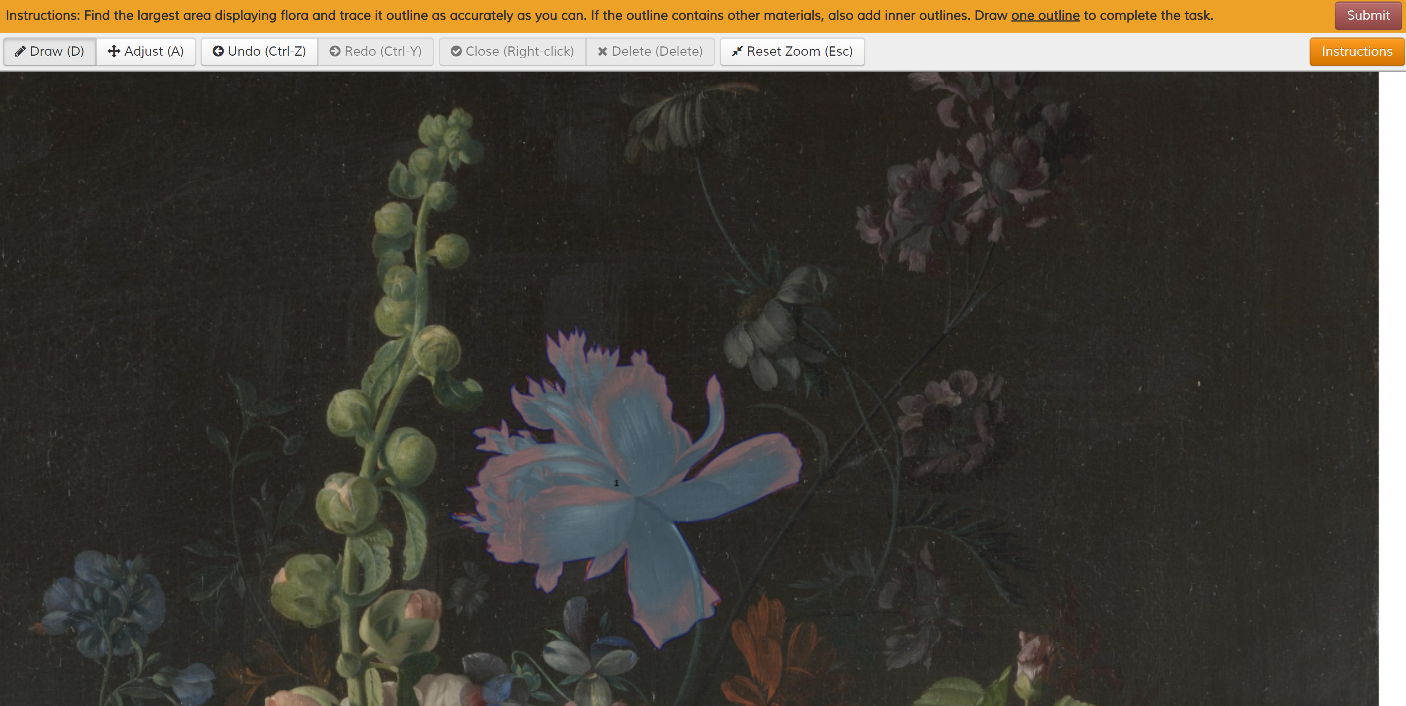


Figure 2. Example of the online segmentation tasks. At the top, a short summary of the instructions is given. Directly below is the menu, which also holds an option to view the instructions again. The remainder of the screen is used to display the painting, which is here displayed zoomed in. In the middle, a flower can be seen, which has been segmented, as indicated with a light-blue transparent layer. Once complete, a participant could press the submit button in the top right.

### Step 4: Quality Check.

In the quality control check a total of 127 AMT participants performed 1349 tasks for 0.05 USD per task. In each task, a participant was shown 20 material segments, together with the paintings the segments were from. This can be seen in figure 3. Participants were asked to inspect each segment and click the painting if it fulfilled the requirements. The requirements were 1) that each segment only contains one material and 2) that the boundaries of the segment follow the outline of the depicted material. Examples were provided. Comparable to the task in step 2, once the segment was clicked, it would get an orange outline and a textbox in the bottom right would change from “multiple materials or bad boundary” to “one material and tight boundary”.
 A minimum of 5 different AMT participants would judge each segment. Next, we did a quality check using the CUBAM algorithm, as described in Bell et al., (2013), which was created by Welinder, Branson, Belongie, & Perona, (2010). CUBAM is intended to improve the quality of binary data by taking the (dis-)agreement between participants into account. The CUBAM algorithm resulted in a single *quality score* value. Segments with an adequate quality score continued to the next step.


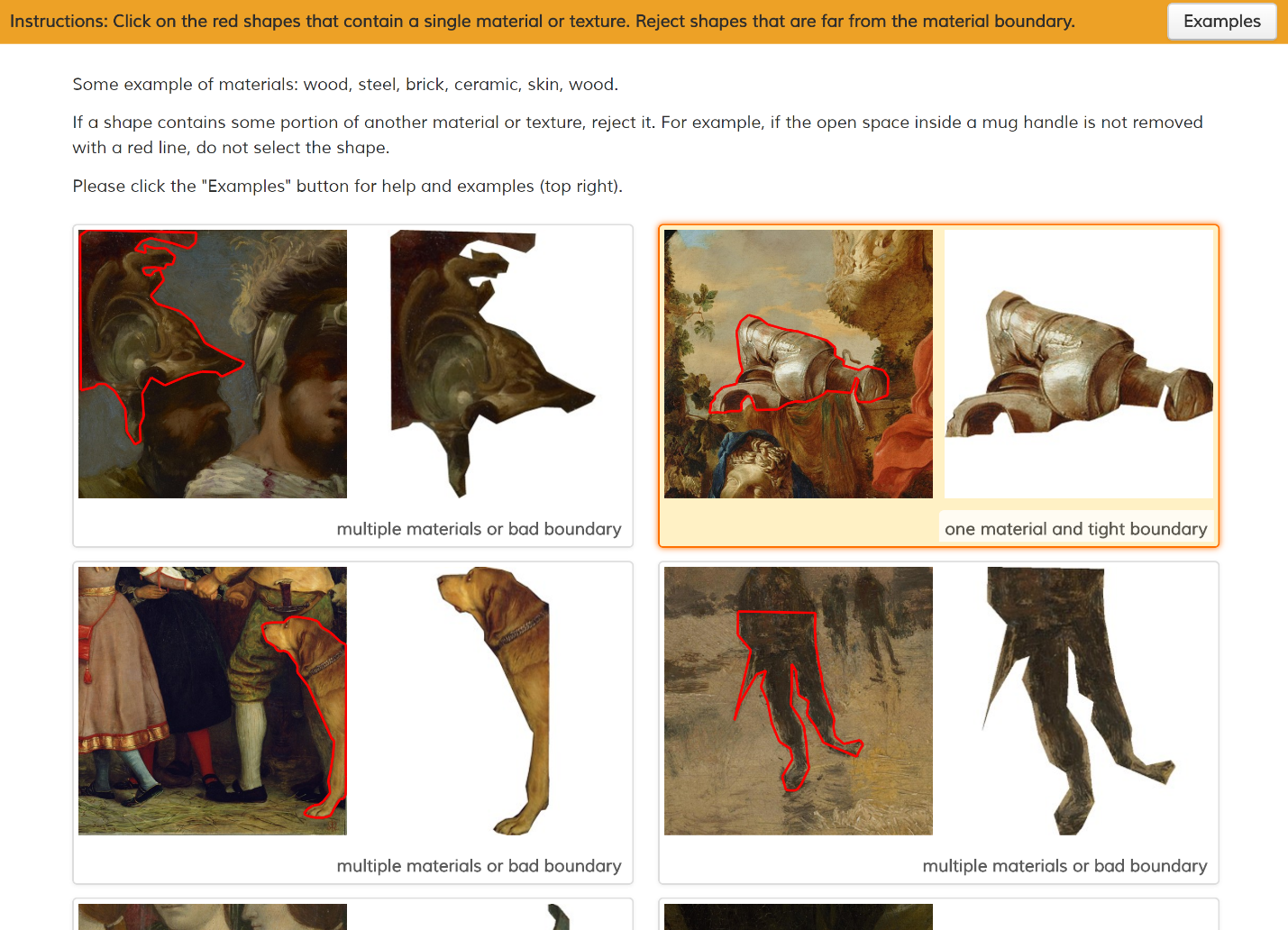


Figure 3. Example of a quality check task. At the top, a summary of the instructions is given, next to a button to see good and bad examples. Segments are displayed next to the section of painting where they are originally taken from, to show the material segment in context. AMT participants could click a pair to indicate the material segmentation was correct, i.e. contained one material and possessed a tight boundary. Participants could scroll down to see the rest of the segments. A submit button was below the last segments.

### Step 5: Material labeling.

In step 3 participants segmented specific materials. It is possible that instead of segmenting the required material a participant segmented a different material. To counter this possibility, in this step we re-labeled each segment with a material name. 178 AMT participants completed 749 of these labeling tasks, for 0.04 USD per task. In each task, 5 different participants were shown one segment at a time and asked to select to best fitting label from a list. The list contained the materials discussed in section *Materials*, as well as three additional options: *“I can’t tell”, “More than one material”* and *“Not on list”*. If there was an agreement on at least 3 out of 5 answers, we assigned that label to the segment.

### Step 6: Manual selection

After completing the previous steps, we had collected around 4500 material segments. From these segments we selected 90 segmentations for each of the 15 material categories, aiming to create the most diverse set possible for each material. These sets were then used for the perceptual experiment.

## Material-specific PCA visualizations


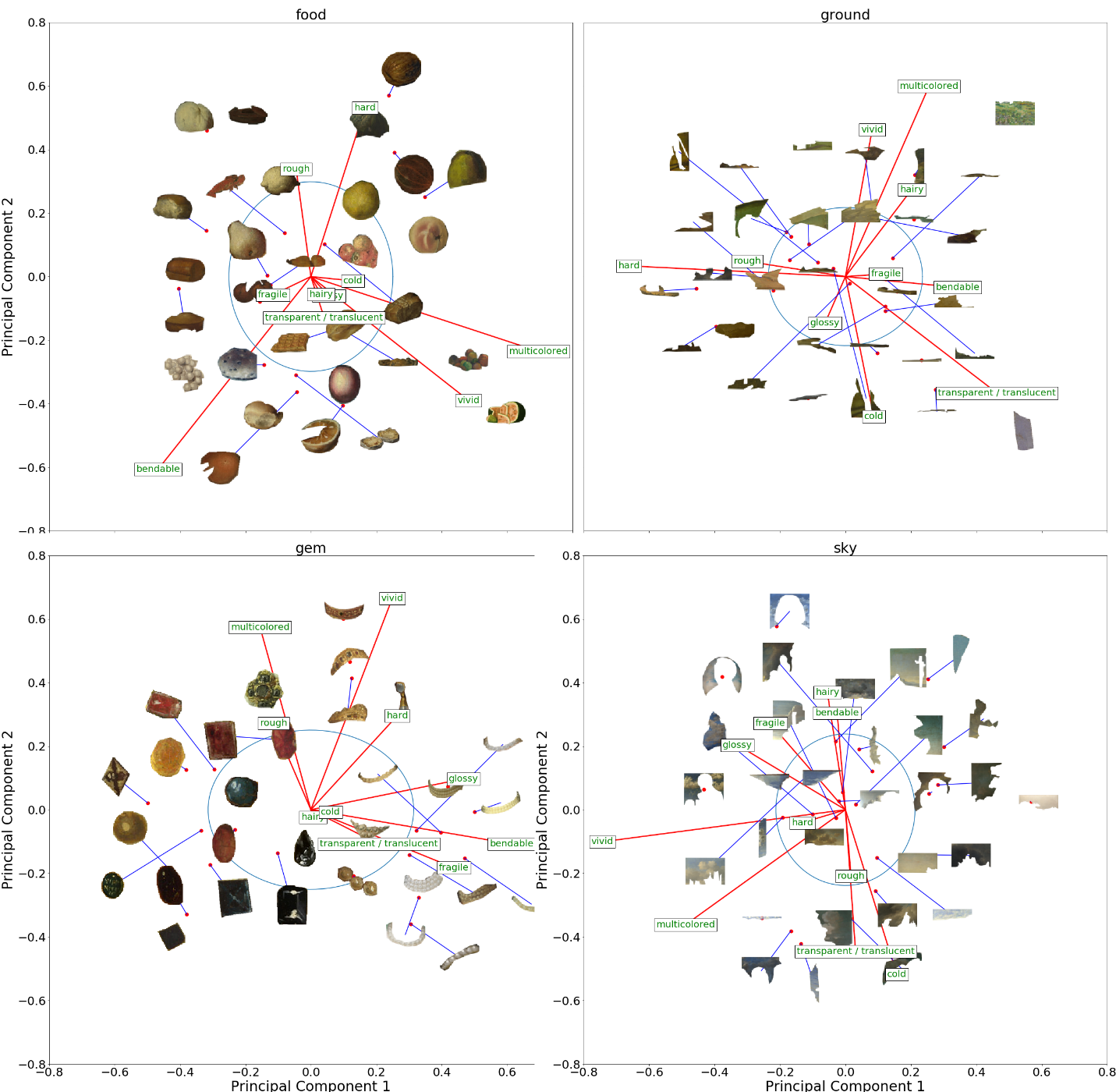


Figure 4. Four visualizations of the first two primary components for the material-specific PCA for food, ground, gem, and sky. Each PCA was run with only the 30 stimuli per material. The red vectors indicate the factor loadings of each attribute. We plotted the actual stimuli within the PCA space. The blue lines connect the stimuli to its actual position within the space when the stimuli would otherwise overlap. The ellipse was fitted around the points (1 sd).


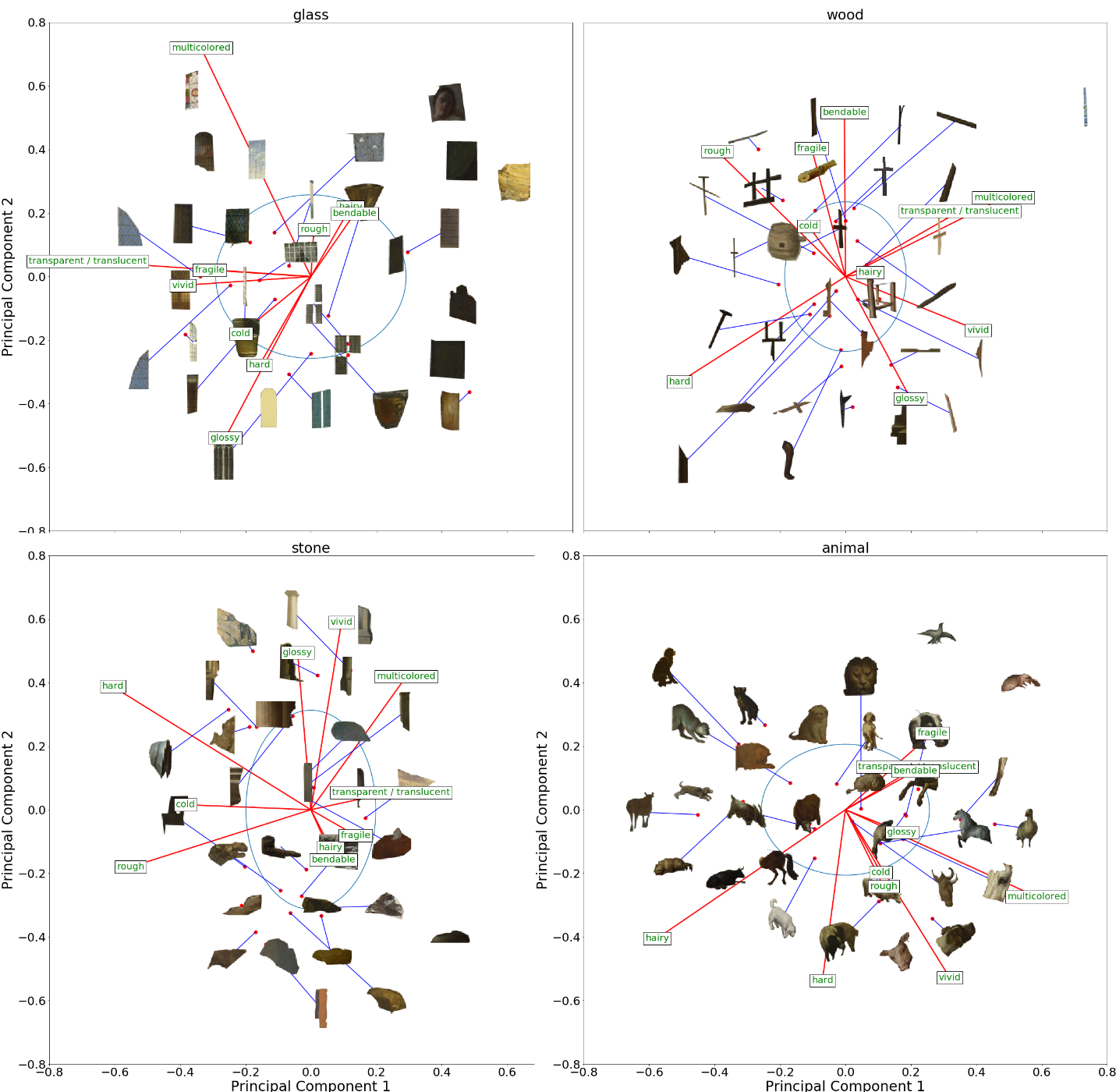


Figure 5. Four visualizations of the first two primary components for the material-specific PCA for glass, wood, stone, and animal. Each PCA was run with only the 30 stimuli per material. The red vectors indicate the factor loadings of each attribute. We plotted the actual stimuli within the PCA space. The blue lines connect the stimuli to its actual position within the space when the stimuli would otherwise overlap. The ellipse was fitted around the points (1 sd).


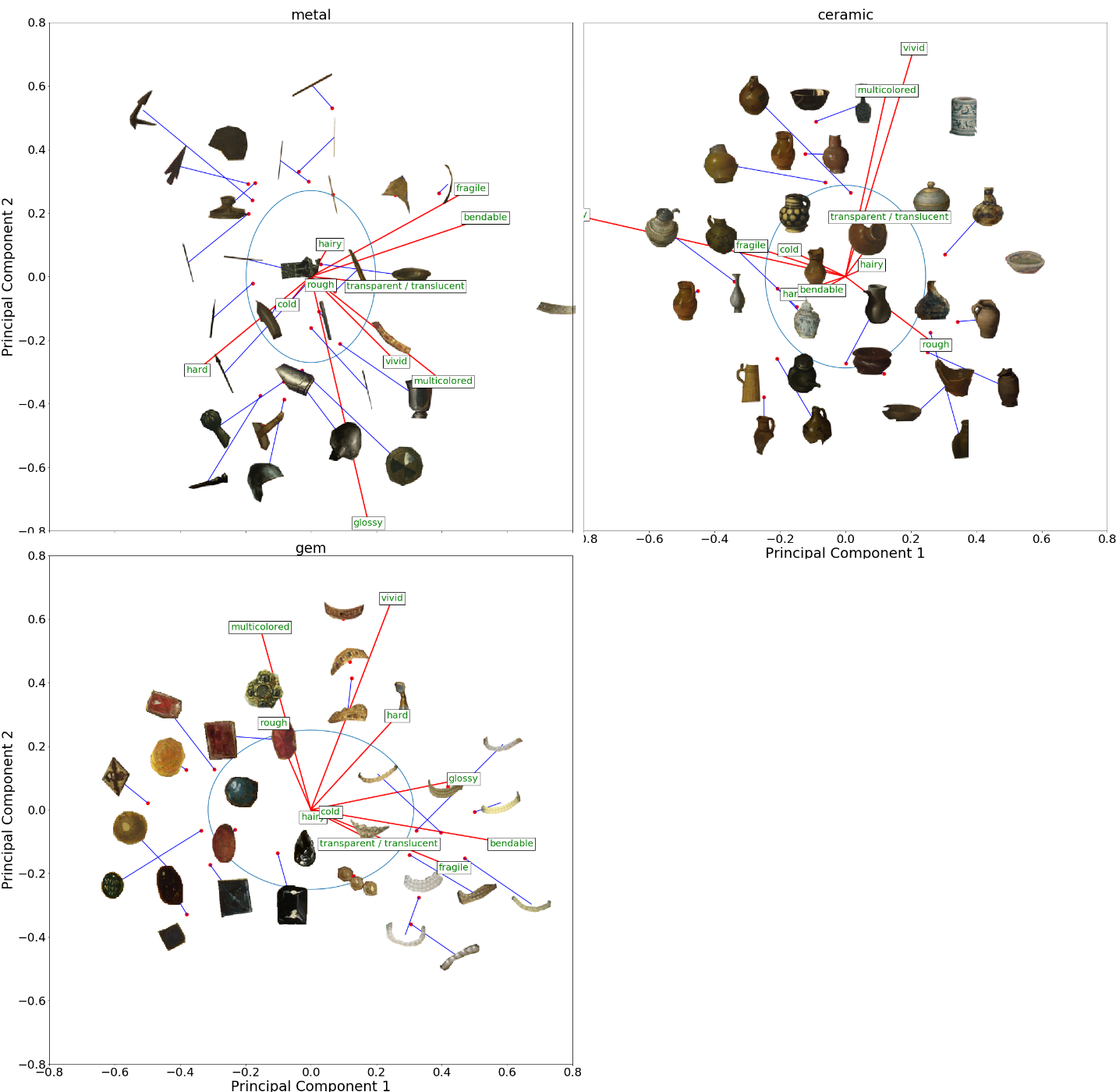


Figure 6. Three visualizations of the first two primary components for the material-specific PCA for metal, ceramic, and gem. Each PCA was run with only the 30 stimuli per material. The red vectors indicate the factor loadings of each attribute. We plotted the actual stimuli within the PCA space. The blue lines connect the stimuli to its actual position within the space when the stimuli would otherwise overlap. The ellipse was fitted around the points (1 sd).

## Factor loadings

| Material |  | Multi-colored | transparent translucent | glossy | hairy | rough | hard | bendable | fragile | cold | vivid |
| --- | --- | --- | --- | --- | --- | --- | --- | --- | --- | --- | --- |
| flora | PC1 | -0.4 | 0.03 | -0.15 | 0.13 | 0.42 | 0.35 | -0.29 | -0.6 | 0.05 | -0.23 |
| flora | PC2 | 0.61 | 0.11 | 0.06 | 0.22 | 0.16 | -0.02 | 0.05 | -0.49 | -0.03 | 0.54 |
| paper | PC1 | -0.36 | -0.1 | 0.08 | -0.15 | 0.22 | 0.45 | -0.61 | -0.32 | 0.09 | -0.31 |
| paper | PC2 | 0.76 | -0.2 | 0.12 | -0.09 | 0.17 | 0.26 | -0.25 | -0.17 | -0.07 | 0.41 |
| glass | PC1 | -0.34 | -0.68 | -0.26 | 0.12 | 0.01 | -0.16 | 0.13 | -0.31 | -0.21 | -0.39 |
| glass | PC2 | 0.72 | 0.05 | -0.51 | 0.22 | 0.15 | -0.28 | 0.2 | 0.02 | -0.18 | -0.03 |
| wood | PC1 | 0.48 | 0.35 | 0.2 | 0.08 | -0.39 | -0.51 | 0 | -0.1 | -0.11 | 0.41 |
| wood | PC2 | 0.25 | 0.21 | -0.38 | 0.01 | 0.39 | -0.33 | 0.52 | 0.4 | 0.16 | -0.17 |
| skin | PC1 | 0.73 | 0.09 | -0.01 | 0.08 | 0.1 | 0.02 | -0.14 | 0.02 | 0.25 | 0.6 |
| skin | PC2 | 0.08 | -0.06 | -0.44 | 0.2 | 0.36 | 0.37 | 0.58 | -0.04 | 0.34 | -0.19 |
| stone | PC1 | 0.29 | 0.25 | -0.04 | 0.06 | -0.55 | -0.61 | 0.07 | 0.14 | -0.38 | 0.09 |
| stone | PC2 | 0.42 | 0.05 | 0.5 | -0.12 | -0.18 | 0.39 | -0.16 | -0.08 | 0.02 | 0.59 |
| animal | PC1 | 0.59 | 0.22 | 0.17 | -0.58 | 0.12 | -0.07 | 0.21 | 0.27 | 0.11 | 0.32 |
| animal | PC2 | -0.27 | 0.14 | -0.07 | -0.4 | -0.24 | -0.54 | 0.12 | 0.24 | -0.2 | -0.53 |
| fabrics | PC1 | -0.46 | -0.16 | -0.34 | 0.05 | 0.19 | 0.25 | -0.22 | -0.11 | 0.2 | -0.67 |
| fabrics | PC2 | 0.41 | -0.09 | 0.19 | -0.53 | 0.22 | 0.39 | -0.27 | -0.21 | 0.43 | 0.07 |
| food | PC1 | 0.7 | 0.04 | 0.05 | 0.03 | -0.05 | 0.17 | -0.47 | -0.12 | 0.13 | 0.48 |
| food | PC2 | -0.24 | -0.13 | -0.06 | -0.06 | 0.34 | 0.53 | -0.61 | -0.06 | -0.01 | -0.39 |
| ground | PC1 | 0.26 | 0.46 | -0.06 | 0.2 | -0.3 | -0.66 | 0.34 | 0.12 | 0.09 | 0.08 |
| ground | PC2 | 0.6 | -0.37 | -0.15 | 0.27 | 0.05 | 0.03 | -0.03 | 0.01 | -0.44 | 0.46 |
| gem | PC1 | -0.16 | 0.21 | 0.47 | 0.01 | -0.11 | 0.26 | 0.61 | 0.44 | 0.06 | 0.25 |
| gem | PC2 | 0.57 | -0.11 | 0.1 | -0.02 | 0.27 | 0.3 | -0.11 | -0.18 | -0.01 | 0.66 |
| sky | PC1 | -0.49 | 0.03 | -0.33 | -0.06 | 0.02 | -0.13 | -0.03 | -0.23 | 0.16 | -0.74 |
| sky | PC2 | -0.36 | -0.45 | 0.2 | 0.37 | -0.21 | -0.04 | 0.31 | 0.27 | -0.52 | -0.1 |
| metal | PC1 | 0.4 | 0.29 | 0.18 | 0.06 | 0.03 | -0.35 | 0.53 | 0.49 | -0.07 | 0.26 |
| metal | PC2 | -0.33 | -0.03 | -0.78 | 0.1 | -0.03 | -0.29 | 0.19 | 0.28 | -0.09 | -0.27 |
| ceramic | PC1 | 0.13 | 0.13 | -0.83 | 0.08 | 0.28 | -0.16 | -0.07 | -0.29 | -0.17 | 0.21 |
| ceramic | PC2 | 0.59 | 0.19 | 0.19 | 0.04 | -0.21 | -0.06 | -0.04 | 0.1 | 0.08 | 0.72 |
| liquid | PC1 | 0.19 | 0.32 | 0.67 | -0.07 | -0.3 | -0.02 | 0.04 | 0.44 | 0.16 | 0.3 |
| liquid | PC2 | -0.35 | -0.2 | 0.08 | -0.08 | 0.13 | 0.44 | -0.02 | 0.62 | -0.38 | -0.29 |
| global | PC1 | -0.13 | -0.08 | 0.09 | -0.27 | 0.22 | 0.62 | -0.56 | -0.12 | 0.33 | -0.17 |
| Global | PC2 | 0.22 | 0.69 | 0.25 | -0.32 | -0.25 | -0.23 | -0.15 | 0.1 | 0.37 | 0.14 |

Table 2. A full list of the factor loadings for the first two principal components for each separate material, and one for all the materials together at the bottom.
